# Use of dipeptidyl peptidase‐4 inhibitors is associated with lower risk of severe renal outcomes in pre‐dialysis patients with Type 2 diabetes

**DOI:** 10.1111/joim.20112

**Published:** 2025-07-03

**Authors:** Tung‐Ying Hung, Tzu‐Chieh Lin, Ying‐Jay Liou, Tzu‐Han Lin, Yu‐Juei Hsu, Liang‐Yu Lin, Meng‐Ting Wang

**Affiliations:** ^1^ Department of Pharmacy National Yang Ming Chiao Tung University Taipei Taiwan; ^2^ Faculty of Medicine National Yang Ming Chiao Tung University Taipei Taiwan; ^3^ Department of Psychiatry Taipei Veterans General Hospital Taipei Taiwan; ^4^ Department of Biochemistry National Defense Medical Center Taipei Taiwan; ^5^ Division of Nephrology Department of Medicine Tri‐Service General Hospital National Defense Medical Center Taipei Taiwan; ^6^ Division of Endocrinology and Metabolism Department of Medicine Taipei Veterans General Hospital Taipei Taiwan; ^7^ College of Medicine National Yang Ming Chiao Tung University Taipei Taiwan

**Keywords:** cohort study, dialysis, dipeptidyl peptidase‐4 inhibitors, end‐stage renal disease, renal replacement therapy

## Abstract

**Objectives:**

Patients with diabetes and Stage 5 chronic kidney disease (CKD) not on dialysis are susceptible to renal replacement therapy and severe complications. Among limited antidiabetic options in this vulnerable population, dipeptidyl peptidase‐4 (DPP‐4) inhibitors (DPP‐4i) are widely used; however, supporting evidence is scant. This study assessed severe renal outcomes associated with DPP‐4i in diabetic and pre‐dialysis patients.

**Methods:**

This study employed an active‐comparator and propensity score–based inverse probability of treatment weighting approach, using Taiwan's nationwide healthcare claims database from 2012 to 2020. We identified patients with diabetes and CKD stage 5 not on dialysis who received erythropoietin (erythropoietin‐stimulating agent), a drug reimbursed for patients with an estimated glomerular filtration rate <15 mL/min/1.73 m^2^. The primary outcome was a composite of renal replacement therapy, renal death, and kidney‐related hospitalization events, and secondary outcomes included each component of the composite and hypoglycemia.

**Results:**

We included 7271 diabetic and pre‐dialysis patients with CKD stage 5, of whom 5028 received DPP‐4i and 2243 received meglitinides. DPP‐4i were associated with a 14% reduced risk of the renal composite outcome compared to meglitinides (weighted hazard ratio [HR], 0.86; 95% confidence interval, 0.81–0.92). Individual component analysis revealed that the decreased risk was confined to renal replacement therapy, with a 17% reduction. DPP‐4i was related to a 41% decreased severe hypoglycemia risk.

**Conclusions:**

In diabetic and pre‐dialysis patients with CKD stage 5, DPP‐4i are related to a lower risk of the renal composite outcome, primarily driven by lower renal dialysis risk, and a lower hypoglycemia risk compared with meglitinides.

## Graphical abstract

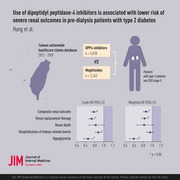

**Abbreviations**
ACEIs: angiotensin‐converting enzyme inhibitors
aDCSI: adapted Diabetes Complications Severity Index
ARBs: angiotensin II receptor blockers
CKD: chronic kidney disease
DPP‐4i: dipeptidyl peptidase‐4 inhibitors
eGFR: estimated glomerular filtration rate
EPO: erythropoietin‐stimulating agent
ESRD: end‐stage renal disease
GERD: gastroesophageal reflux disease
GIP: gastric inhibitory peptide
GLP‐1 RA: glucagon‐like peptide‐1 receptor agonists
HR: hazard ratio
IPTW: inverse probability of treatment weighting
KDIGO: Kidney Disease: Improving Global Outcomes
NHI: National Health Insurance
NNT: number needed to treat
PPV: positive predictive value
PS: propensity score
RCTs: randomized controlled trials
SGLT2i: sodium–glucose cotransporter‐2 inhibitors
SMD: standardized mean difference
T2DM: Type 2 diabetes mellitus

## Introduction

Diabetic nephropathy is the leading cause of chronic kidney disease (CKD) [1], with approximately 40% of the 537 million diabetic patients worldwide developing CKD [2, 3] and potentially progressing to CKD stage 5 or even end‐stage renal disease (ESRD), which ultimately requires dialysis or kidney transplantation [4]. The presence of ESRD in patients with diabetes poses a significant global health threat, leading to increased mortality, deteriorated quality of life, and high healthcare costs [5, 6, 7]. Therefore, preventing the progression of severe CKD to dialysis, renal transplantation, or death is critically important.

The choices of antidiabetic agents for patients with diabetes and CKD stage 5 are limited, and dose adjustment is often required, primarily due to severely decreased renal function, the renal elimination of most antidiabetic drugs, and the risk of drug‐induced hypoglycemia. Although sodium–glucose cotransporter‐2 inhibitors (SGLT2i) have shown renal protective effects [8, 9, 10], initiation of SGLT2i is not recommended in Type 2 diabetes mellitus (T2DM) patients with estimated glomerular filtration rate (eGFR) under 20 mL/min/1.73 m^2^ because these drugs can cause a drop in eGFR during early treatment and have reduced glucose‐lowering effects in advanced CKD [11, 12]. Similarly, glucagon‐like peptide‐1 receptor agonists (GLP‐1 RAs), another newer class of antidiabetic medications, have been demonstrated to have renal protective effects in randomized controlled trials (RCTs) [13, 14]. However, GLP‐1 RA use in patients with diabetes and pre‐dialysis is relatively uncommon [15], primarily due to intolerance of gastrointestinal side effects—such as nausea, vomiting, and diarrhea—in this vulnerable population [11]. Consequently, patients with diabetes and CKD stage 5 have restricted choices of oral antidiabetic medications with approved reno‐protective effects.

Dipeptidyl peptidase‐4 inhibitors (DPP‐4i) are widely used in clinical practice and recommended by the current Kidney Disease: Improving Global Outcomes (KDIGO) guidelines for patients with severe renal impairment [11]; however, the corresponding supporting evidence is limited. In RCTs that excluded pre‐dialysis patients with an eGFR <15, DPP‐4i have been shown to reduce albuminuria risk [16, 17]. Specifically, the CARMELINA trial reported a 14% reduction in albuminuria with the use of linagliptin in patients at high cardiovascular and renal risk [16]. Similarly, a post hoc analysis of SAVOR‐TIMI 53 found that saxagliptin improved or stabilized the albumin‐to‐creatinine ratio in patients with diabetes across albuminuria stages [17]. A meta‐analysis of 23 RCTs observed 11% and 23% reductions in microalbuminuria and macroalbuminuria risk, respectively, when excluding SGLT2 inhibitors in the control arm [18]. However, all these RCTs excluded pre‐dialysis patients with T2DM due to ethical concerns, leaving the impact of DPP‐4i on renal outcomes in CKD stage 5 patients not receiving dialysis uncertain.

This study aimed to investigate whether use of DPP‐4i is related to a reduced risk of a composite renal outcome, including dialysis or renal transplantation, hospitalization for kidney‐related events, and renal‐specific deaths in a population of pre‐dialysis patients with T2DM in real‐world clinical settings.

## Methods

### Data source

We conducted an active‐comparator, propensity score (PS)‐based inverse probability of treatment weighting (IPTW) cohort study using Taiwan's National Health Insurance (NHI) claims database from 2012 to 2020. Since the implementation of Taiwan's universal NHI in 1996, the NHI claims database has captured comprehensive medical care claims, including ambulatory, emergency care, and inpatient services, as well as prescribed medications and prescriptions filled at pharmacies, covering over 99% of Taiwanese inhabitants [19]. Various disease codes have been confirmed with high accuracy in the NHI database, making it a reliable source for assessing drug effectiveness and safety [20]. We linked the NHI claims database to the nationwide death registry for assessing mortality data and to the catastrophic illness file, containing detailed data on nearly all patients undergoing dialysis for at least 3 months, to accurately identify patients on dialysis. Patients receiving dialysis for ≥3 months are eligible for the catastrophic illness program, which waives medical copayments and requires approval from two nephrologists. All data from the NHI claims and death registry are double‐encrypted. This study was approved by the National Yang Ming Chiao Tung University (approval number: YM111121W). Informed consent was waived because deidentified data were analyzed. This study was presented following the Strengthening the Reporting of Observational Studies in Epidemiology (STROBE) reporting guideline.

### Cohort identification

We first identified patients with diabetes mellitus and CKD stage 5 who first received erythropoietin‐stimulating agent (EPO) as the base study cohort between 2013 and 2019. In Taiwan, under the NHI reimbursement policy, EPO is reimbursed for patients having CKD with an eGFR <15 mL/min/1.73 m^2^, which was used to identify patients with Stage 5 CKD in this study. The surrogate definition of CKD stage 5 has a reported positive predictive value of 95.9% [21]. Additionally, over 70% of patients with diabetes and CKD stage 5 receive EPO [15, 22]. We required patients to have at least two outpatient visits or one inpatient visit in 1 year for diabetes and CKD, respectively. The coding algorithms for the two diseases have been found to be highly accurate [21, 23, 24].

From the base cohort, we required patients to receive either DPP‐4i or meglitinide after their first EPO prescription between 2013 and 2019. The date of the first DPP‐4i or meglitinide prescription after EPO initiation marked the cohort entry. Meglitinides were chosen as the comparator because (1) they were the second most frequently used oral antidiabetic medication after DPP‐4i based on our pilot data in CKD stage 5 patients, and (2) no known associations between meglitinides and renal outcomes have been reported [25, 26]. After the first EPO use, we included new users—those with no DPP‐4i or meglitinide use in the prior 6 months—and persistent combination therapy users, who initially received both DPP‐4i and meglitinides before EPO initiation but switched to monotherapy after EPO use. Persistent users were included to avoid loss of sample size when adopting a new‐user design, as initiating a new antidiabetic therapy is rare in this vulnerable population with diabetes and severe CKD. To minimize prevalent user bias among persistent users, we accounted for the duration of prior dual combination therapy before the initiation of EPO. Patients had to be aged ≥18 years on the cohort entry date. Patients meeting these criteria formed the study cohort.

We excluded patients receiving maintenance dialysis or kidney transplantation, having diagnoses of Type 1 diabetes mellitus or gestational diabetes, or having less than 1 year of NHI coverage prior to cohort entry. Additionally, patients hospitalized for CKD in the 60 days preceding cohort entry or those using both DPP‐4i and meglitinides on the cohort entry date were also excluded. The study cohort was followed using an on‐treatment exposure definition, requiring continuous treatment during follow‐up. Continuous treatment was defined as the duration of a prescription plus a 60‐day grace period overlapping with the next prescription date. The cohort was followed from the cohort entry date until any of the following events occurred: renal composite outcome (defined below), switching to or adding a study drug, treatment discontinuation, termination of NHI enrollment, death, or study end (December 31, 2020). Operational definitions of selection criteria are detailed in Table S1.

### Outcome measurement

The primary outcome was time‐to‐first renal composite event, including renal replacement (dialysis or kidney transplantation), renal death (based on the primary and secondary diagnosis position in the death registry), and hospitalization with a primary discharge code of kidney‐related events [27, 28]. Renal replacement and renal death were determined from the catastrophic illness file and the national death registry, respectively, ensuring accurate measurement of these outcomes. Secondary outcomes included individual components of the primary outcome, 3‐point major adverse cardiovascular events (3P‐MACE: hospitalization for myocardial infarction, ischemic stroke, or cardiovascular death), hospitalization for heart failure, all‐cause mortality, and hypoglycemia. Diagnosis codes for heart failure, myocardial infarction, ischemic stroke, and hypoglycemia are highly accurate [29, 30, 31, 32, 33, 34]. Table S1 provides the corresponding ICD‐9 codes and ICD‐10 codes along with the relevant procedure definitions.

### Potential confounders

Multiple dimensions of potential confounders measured before or on cohort entry were considered, including demographics (age, sex, cohort entry year, and monthly income‐based insurance premiums as a proxy for socioeconomic status). Renal disease severity was assessed using proxies such as time from EPO initiation to cohort entry, acute kidney disease, proteinuria, and edema. Healthcare utilization measures (e.g., kidney‐related outpatient and inpatient visits and numbers of Hemoglobin A1c [HbA1c] and serum creatinine test orders) and proxies of diabetes severity (e.g., complexity of antidiabetic treatment, type of antidiabetic medications, and the adapted Diabetes Complications Severity Index [aDCSI]) [35] were included. Comorbidities (e.g., anemia and cardiovascular and pulmonary diseases) and comedications (e.g., angiotensin‐converting enzyme inhibitors [ACEIs], angiotensin II receptor blockers [ARBs], and pentoxifylline) were also measured. Demographic data were collected at cohort entry, whereas antidiabetic treatments were evaluated both during the 6 months before and at cohort entry. Other comedications and confounders were assessed in the 6 months and the year preceding cohort entry, respectively. Operational definitions of the confounders are shown in Table S1.

### Sensitivity analyses and subgroup analyses

Predetermined sensitivity analyses were performed to evaluate the robustness of our findings. To address potential informative censoring bias, we conducted a 1‐year intention‐to‐treat approach, irrespective of treatment changes or discontinuation. Treatment continuation was redefined using 30‐ and 90‐day grace periods. To reduce misclassification of kidney‐related deaths, only primary kidney‐related deaths were included. Non‐renal deaths, which could compete with the composite renal outcome, were treated as competing events using the Fine and Gray's approach [36]. PS trimming was applied to exclude nonoverlapping regions between groups to reduce the impact of extreme weights in the used weighting approach. The weighting approach was detailed in the Statistical Analysis section. Additionally, we performed a multiple Cox regression model adjusted for unbalanced characteristics at baseline as an alternative to IPTW to mitigate confounding effects on our findings while preserving the original sample size of the study cohort. We additionally excluded patients with cancer diagnoses, as the NHI reimbursement policy also allows EPO to be used for cancer patients. We also restricted DPP‐4i uses to linagliptin users only, as it is the only DPP‐4i shown to reduce the risk of albuminuria in the RCTs. Lastly, analyses were repeated using a 1:1 PS matching and a new‐user design.

To evaluate potential effect modifiers, we conducted stratified analyses based on exposure status (new‐user vs. persistent‐user), enrollment in Taiwan's pre‐ESRD care program, prior insulin use, history of renal event hospitalization, cardiovascular disease history, prior use of ACEI or ARBs, and the mean of aDCSI. The pre‐ESRD program is a universal pay‐for‐performance program that has been aiming to adopt multidisciplinary care for CKD stage 3b‐5 patients in Taiwan since 2006.

### Additional analysis

To address unmeasured confounding, gastroesophageal reflux disease (GERD) was used as a negative control outcome, given its association with diabetes mellitus severity but not the drugs of interest [37]. Additionally, we estimated the *E*‐value to examine the potential influence of an unmeasured confounder on the primary renal composite outcome [38].

### Statistical analyses

Patient characteristics between the DPP‐4i and meglitinide groups were compared, with standardized mean differences (SMDs) exceeding 10% indicating meaningful differences. PS‐based IPTW was used to maintain comparability between the two groups [39]. PSs—the probabilities of receiving DPP‐4i—were estimated using a multiple logistic regression model with predefined confounders as predictors [40]. We assigned weights of 1/PS for the DPP‐4 inhibitor group and 1/(1‐PS) for the meglitinide group, stabilizing the weights with the marginal probability of receiving DPP‐4i [41]. Weighted Cox proportional hazards models were used to estimate hazard ratios (HRs) for the composite renal outcome and secondary outcomes for DPP‐4i use versus meglitinide use. Sandwich robust variance estimators were applied for all Cox proportional hazards models. The proportionality assumption was examined by assessing Schoenfeld residuals, and all models met this assumption [42] (Table S2). Accumulated incidences of the outcomes were quantified for each group using a weighted Kaplan–Meier approach, and incidences were compared between the two groups using log‐rank tests. The number needed to treat (NNT) for outcomes with significant differences was calculated using the difference in weighted cumulative incidence between the groups during a 1‐year period [43]. For the sample size calculation, this study required 1946–3926 patients in each group to detect a 20% reduction in the risk of renal outcomes in DPP‐4i versus meglitinides, with 80% statistical power, based on the findings from the CARMELINA trial (details in “Methods” section in Supporting Information file). All tests were two‐tailed, with *p*‐values <0.05 indicating statistical significance. Analyses were conducted using SAS, version 9.4 (SAS Institute Inc., Cary, NC, USC), and Stata, version 16 (StataCorp LLC).

## Results

After applying the inclusion and exclusion criteria, 7271 patients with T2DM and CKD stage 5 were identified, including 5028 patients receiving DPP‐4i and 2243 patients receiving meglitinides (Fig. S1). Following the IPTW application, we analyzed 5027 DPP‐4i users and 2241 meglitinide users (Table 1). The overall mean age (standard deviation [SD]) between the two groups was 68.7 (12.30), and approximately 50% of the study cohort were male. The full baseline characteristics of DPP‐4 inhibitor users versus meglitinide users before and after IPTW are shown in Table S3. The overall mean (SD) follow‐up duration in days between the two groups was 132.6 (223.1). Most patients were censored during follow‐up due to the occurrence of outcome (DPP‐4i group: 65.4%; meglitinides: 64.3%), and other reasons for censoring are provided in Table S4, with <6% of the SMDs between the two groups.

**Table 1:** Selected baseline characteristics of dipeptidyl peptidase 4 (DPP‐4) inhibitor users versus meglitinide users before and after inverse probability of treatment weighting (IPTW).

	Before IPTW			After IPTW		
Characteristics	DPP‐4 inhibitor *n* = 5028	Meglitinide *n* = 2243	SMD	DPP‐4 inhibitor *n* = 5027	Meglitinide *n* = 2241	SMD
**Demographics, no. (%)**						
Age, mean (SD)^a^	68.78 (12.34)	68.51 (12.21)	0.02	68.69 (12.34)	68.76 (12.18)	−0.01
Sex (male)	2508 (49.88)	1152 (51.36)	0.09	2525 (50.22)	1122 (50.05)	0.01
Cohort entry year						
2013	295 (5.87)	335 (14.94)	−0.30	435 (8.65)	194 (8.65)	0.00
2014	466 (9.27)	398 (17.74)	−0.25	597 (11.88)	268 (11.94)	0.00
2015	556 (11.06)	319 (14.22)	−0.10	607 (12.07)	271 (12.07)	0.00
2016	774 (15.39)	329 (14.67)	0.02	760 (15.11)	336 (15.00)	0.00
2017	897 (17.84)	330 (14.71)	0.08	851 (16.94)	388 (17.32)	−0.01
2018	999 (19.87)	289 (12.88)	0.19	889 (17.69)	395 (17.61)	0.00
2019	1041 (20.70)	243 (10.83)	0.27	888 (17.66)	390 (17.42)	0.01
**Measures of healthcare utilization, no (%)**						
Hospitalizations						
Kidney‐related						
0	4324 (86.00)	1878 (83.73)	0.06	4284 (85.22)	1905 (85.00)	0.01
1	586 (11.65)	285 (12.71)	−0.03	603 (11.99)	273 (12.19)	−0.01
2+	118 (2.35)	80 (3.57)	−0.07	140 (2.79)	63 (2.81)	0.00
DM‐related						
0	4522 (89.94)	2037 (90.82)	−0.03	4532 (90.16)	2018 (90.02)	0.00
1	451 (8.97)	186 (8.29)	0.02	442 (8.79)	200 (8.93)	−0.01
2+	55 (1.09)	20 (0.89)	0.02	53 (1.05)	23 (1.04)	0.00
CV‐related						
0	3,848 (76.53)	1,661 (74.05)	0.06	3815 (75.89)	1708 (76.19)	−0.01
1	829 (16.49)	396 (17.65)	−0.03	842 (16.74)	366 (16.33)	0.01
2+	351 (6.98)	186 (8.29)	−0.05	370 (7.37)	168 (7.48)	0.00
Other						
0	2937 (58.41)	1331 (59.34)	−0.02	2951 (58.69)	1321 (58.93)	0.00
1	1223 (24.32)	529 (23.58)	0.02	1213 (24.13)	547 (24.40)	−0.01
2+	868 (17.26)	383 (17.08)	0.01	863 (17.18)	374 (16.67)	0.01
Pre‐ESRD program	2319 (46.12)	990 (44.14)	0.04	2289 (45.54)	1022 (45.61)	0.00
**Proxy indicators of DM severity, no (%)**						
Exposure status						
New users	3697 (73.53)	1674 (74.63)	−0.03	3713 (73.86)	1646 (73.45)	0.01
Persistent combination therapy users	1331 (26.47)	569 (25.37)	0.03	1314 (26.14)	595 (26.55)	−0.01
Duration of previous therapy, mean days (SD)^a^	90.20 (258.82)	87.23 (248.44)	0.01	89.16 (257.64)	89.97 (248.77)	0.00
**DM medication within 180 days prior to entry date**						
TZD	410 (8.15)	129 (5.75)	0.09	372 (7.39)	164 (7.30)	0.00
GLP‐1 RA	62 (1.23)	25 (1.11)	0.01	61 (1.21)	29 (1.29)	−0.01
Insulin	3018 (60.02)	1276 (56.89)	0.06	2978 (59.24)	1334 (59.54)	−0.01
Biguanides	574 (11.42)	231 (10.30)	0.04	554 (11.03)	251 (11.21)	−0.01
AGI	630 (12.53)	279 (12.44)	0.00	628 (12.49)	284 (12.69)	−0.01
SGLT2i	38 (0.76)	8 (0.36)	0.05	32 (0.64)	18 (0.82)	−0.02
aDCSI, mean(SD)^a^	4.41 (2.34)	4.24 (2.38)	0.07	4.35 (2.37)	4.34 (2.32)	0.01
Hypoglycemia	725 (14.42)	291 (12.97)	0.04	703 (13.98)	311 (13.89)	0.00
**Proxy indicators of renal severity, no (%)**						
Periods from EPO initiation to cohort entry, mean days (SD)^a^	130.10 (218.55)	112.21 (185.73)	0.09	124.72 (210.66)	123.12 (201.75)	0.01
Acute kidney disease	1083 (21.54)	468 (20.86)	0.02	1075 (21.38)	476 (21.23)	0.00
Proteinuria	171 (3.40)	91 (4.06)	−0.03	181 (3.61)	87 (3.88)	−0.01
Disorders of electrolyte	957 (19.03)	468 (20.86)	−0.05	993 (19.76)	460 (20.52)	−0.02
Disorders of fluid balance	148 (2.94)	84 (3.74)	−0.04	161 (3.20)	71 (3.17)	0.00
Edema	501 (9.96)	330 (14.71)	−0.14	570 (11.34)	252 (11.24)	0.00
Kidney and urinary stone	191 (3.80)	75 (3.34)	0.02	187 (3.72)	89 (3.96)	−0.01
**Comorbidities, no (%)**						
**Cardiovascular disease**						
Myocardial infarction	296 (5.89)	127 (5.66)	0.01	290 (5.76)	129 (5.74)	0.00
Ischemic stroke	553 (11.00)	261 (11.64)	−0.02	561 (11.15)	249 (11.13)	0.00
Hemorrhage stroke	77 (1.53)	28 (1.25)	0.02	71 (1.40)	27 (1.21)	0.02
Other stroke	677 (13.46)	302 (13.46)	0.00	677 (13.47)	303 (13.54)	0.00
Heart failure	1,427 (28.38)	624 (27.82)	0.01	1410 (28.04)	613 (27.33)	0.02
Arrhythmia	402 (8.00)	192 (8.56)	−0.02	411 (8.17)	182 (8.10)	0.01
Peripheral vascular disease	189 (3.76)	97 (4.32)	−0.03	199 (3.96)	91 (4.07)	−0.01
Venous thromboembolism	56 (1.11)	32 (1.43)	−0.03	63 (1.25)	27 (1.20)	0.00
Coronary revascularization	193 (3.84)	82 (3.66)	0.01	190 (3.77)	85 (3.77)	0.00
Hypertension	4452 (88.54)	2012 (89.70)	−0.04	4469 (88.91)	1998 (89.12)	−0.01
Dyslipidemia	2344 (46.62)	1025 (45.70)	0.02	2330 (46.35)	1048 (46.78)	−0.01
**Neurologic disorders**						
Dementia	341 (6.78)	140 (6.24)	0.02	333 (6.63)	157 (7.01)	−0.02
Epilepsy	72 (1.43)	40 (1.78)	−0.03	76 (1.51)	33 (1.47)	0.00
**Other diseases**						
Anemia	1756 (34.92)	857 (38.21)	−0.07	1804 (35.90)	809 (36.12)	0.00
Thyroid disease	194 (3.86)	69 (3.08)	0.04	184 (3.66)	84 (3.76)	−0.01
Liver disease	472 (9.39)	189 (8.43)	0.03	460 (9.14)	213 (9.49)	−0.01
GERD	599 (11.91)	243 (10.83)	0.03	581 (11.55)	257 (11.48)	0.00
Autoimmune diseases	215 (4.28)	95 (4.24)	0.00	213 (4.24)	92 (4.12)	0.01
**Comedications, no (%)**						
Cardiovascular system drugs						
ACEIs	585 (11.63)	262 (11.68)	0.00	585 (11.63)	262 (11.71)	0.00
ARBs	3038 (60.42)	1270 (56.62)	0.08	2978 (59.25)	1339 (59.73)	−0.01
α‐Agonists	42 (0.84)	25 (1.11)	−0.03	47 (0.94)	20 (0.91)	0.00
α‐Blockers	1314 (26.13)	614 (27.37)	−0.03	1329 (26.44)	592 (26.42)	0.00
β‐Blockers	3101 (61.67)	1418 (63.22)	−0.03	3128 (62.22)	1403 (62.58)	−0.01
Calcium channel blockers						
Dihydropyridines	4194 (83.41)	1919 (85.56)	−0.06	4225 (84.05)	1886 (84.13)	0.00
Non‐dihydropyridines	401 (7.98)	209 (9.32)	−0.05	419 (8.34)	183 (8.18)	0.01
Diuretics						
Loop	3717 (73.93)	1719 (76.64)	−0.06	3763 (74.85)	1679 (74.91)	0.00
Thiazides	1535 (30.53)	718 (32.01)	−0.03	1565 (31.14)	709 (31.63)	−0.01
Potassium‐sparing agents	696 (13.84)	297 (13.24)	0.02	687 (13.67)	309 (13.80)	0.00
Lipid‐lowering agents						
Statins	2763 (54.95)	1168 (52.07)	0.06	2717 (54.05)	1212 (54.06)	0.00
Non‐statin agents	440 (8.75)	171 (7.62)	0.04	421 (8.38)	188 (8.39)	0.00
Antiplatelets	2635 (52.41)	1,169 (52.12)	0.01	2630 (52.32)	1179 (52.62)	−0.01
Anticoagulants	820 (16.31)	348 (15.51)	0.02	808 (16.07)	353 (15.76)	0.01
Nitrates	1666 (33.13)	750 (33.44)	−0.01	1670 (33.21)	742 (33.08)	0.00
Antiarrhythmics	447 (8.89)	200 (8.92)	0.00	445 (8.84)	192 (8.57)	0.01
Digoxin	126 (2.51)	67 (2.99)	−0.03	135 (2.68)	60 (2.66)	0.00
Other medication						
Pentoxifylline	1918 (38.15)	785 (35.00)	0.07	1874 (37.28)	837 (37.35)	0.00
Ketosteril	889 (17.68)	289 (12.88)	0.13	814 (16.20)	354 (15.77)	0.01
Proton pump inhibitors	1348 (26.81)	550 (24.52)	0.05	1309 (26.04)	580 (25.86)	0.00
Drugs for hyperkalemia and hyperphosphatemia	2294 (45.62)	1120 (49.93)	−0.09	2356 (46.86)	1056 (47.10)	0.00

Abbreviations: ACEIs, angiotensin‐converting enzyme inhibitors; aDCSI, adapted Diabetes Complication Index; AGI, α‐glucosidase inhibitor; ARBs, angiotensin II receptor blockers; CV, cardiovascular; DM, diabetes mellitus; DPP‐4 inhibitor, dipeptidyl peptidase 4 inhibitor; EPO, erythropoietin‐stimulating agent; ESRD, end‐stage renal disease; GERD, gastroesophageal reflux disease; GLP‐1 RA, glucagon‐like peptide‐1 receptor agonists; SD, standard deviation; SGLT2i, sodium–glucose cotransporter‐2 inhibitor; SMD, standardized mean difference; TZD, thiazolidinedione.

^a^ Continuous variables were treated as restricted cubic splines with five knots in the logistic regression model estimating the propensity scores, except for the time period from EPO initiation to cohort entry, which was treated as restricted cubic splines with three knots.

Before IPTW was applied, the majority of baseline characteristics—including proxy indicators for diabetes and renal disease severity, cardiovascular diseases and other comorbidities, and comedications—were all balanced, except for cohort entry year, edema, and use of ketosteril. However, all baseline characteristics were well balanced between the DPP‐4i and meglitinide groups after IPTW (Table 1).

Table 2 presents the comparative risks of the examined outcomes. During the follow‐up, a total of 3286 and 1442 events of composite renal outcomes occurred among DPP‐4i and meglitinide users, respectively. The weighted incidence rate was 162.0 per 100 person‐years in DPP‐4i users, compared with 251.6 in meglitinide users. The cumulative incidence rates for all examined outcomes are shown in Figs. S2 and S3. DPP‐4i versus meglitinide use was related to a 14% reduced risk of the composite renal outcome, which included renal replacement, renal death, and hospitalization for kidney‐related events (weighted HR, 0.86; 95% confidence interval [CI], 0.81–0.92), with an NNT of 11 (Table S5). In the secondary outcomes, the use of DPP‐4i versus meglitinide was associated with a reduced risk of renal replacement (weighted HR, 0.83; 95% CI, 0.77–0.90); however, a non‐significant lower risk was observed for renal death (weighted HR, 0.82; 95% CI, 0.66–1.03), hospitalizations of kidney‐related events (weighted HR, 0.95; 95% CI, 0.88–1.03), and all‐cause mortality (weighted HR, 0.85; 95% CI, 0.71–1.01). In addition, no significant difference was observed in risks of 3P‐MACE (weighted HR, 0.90; 95% CI, 0.73–1.10) and hospitalization of HF (weighted HR, 1.04; 95% CI, 0.79–1.36). As for the risk of severe hypoglycemia, DPP‐4i was associated with a 41% decreased risk compared to meglitinide (weighted HR, 0.59; 95% CI, 0.49–0.70).

**Table 2:** Comparative risk of renal and cardiovascular events between dipeptidyl peptidase‐4 inhibitor (DPP‐4i) users and meglitinide users.

	DPP‐4i (*N* = 5028)			Meglitinide (*N* = 2243)				
		IR (100 person‐years)			IR (100 person‐years)		HR (95% CI)	
Outcomes	Events	Crude	Weighted	Events	Crude	Weighted	Crude	Weighted
**Primary composite outcome**	3286	155.2	162.0	1442	276.0	251.6	0.78 (0.73–0.83)^*^	0.86 (0.81–0.92)^*^
**Secondary outcomes**								
Renal replacement therapy	2318	88.7	92.3	976	146.5	131.8	0.73 (0.67–0.79)^*^	0.83 (0.77–0.90)^*^
Renal death	325	6.3	6.3	128	9.1	9.3	0.82 (0.67–1.01)	0.82 (0.66–1.03)
Hospitalization of kidney‐related events	2323	78.3	80.3	984	120.8	116.4	0.88 (0.82–0.95)^*^	0.95 (0.88–1.03)
3‐point MACE	412	8.2	8.0	145	10.5	11.1	0.94 (0.78–1.14)	0.90 (0.73–1.10)
Hospitalization of heart failure	200	4.0	4.1	86	6.3	5.6	0.85 (0.66–1.10)	1.04 (0.79–1.36)
All‐cause mortality	509	9.9	9.8	190	13.4	14.1	0.87 (0.73–1.03)	0.85 (0.71–1.01)
Hypoglycemia	376	7.7	7.3	212	16.2	17.7	0.65 (0.55–0.77)^*^	0.59 (0.49–0.70)^*^

Abbreviations: 3‐point MACE, 3‐point major adverse cardiovascular events; CI, confidence interval; HR, hazard ratio; IR, incidence rate.

* *p* < 0.05.

**Fig. 1:**
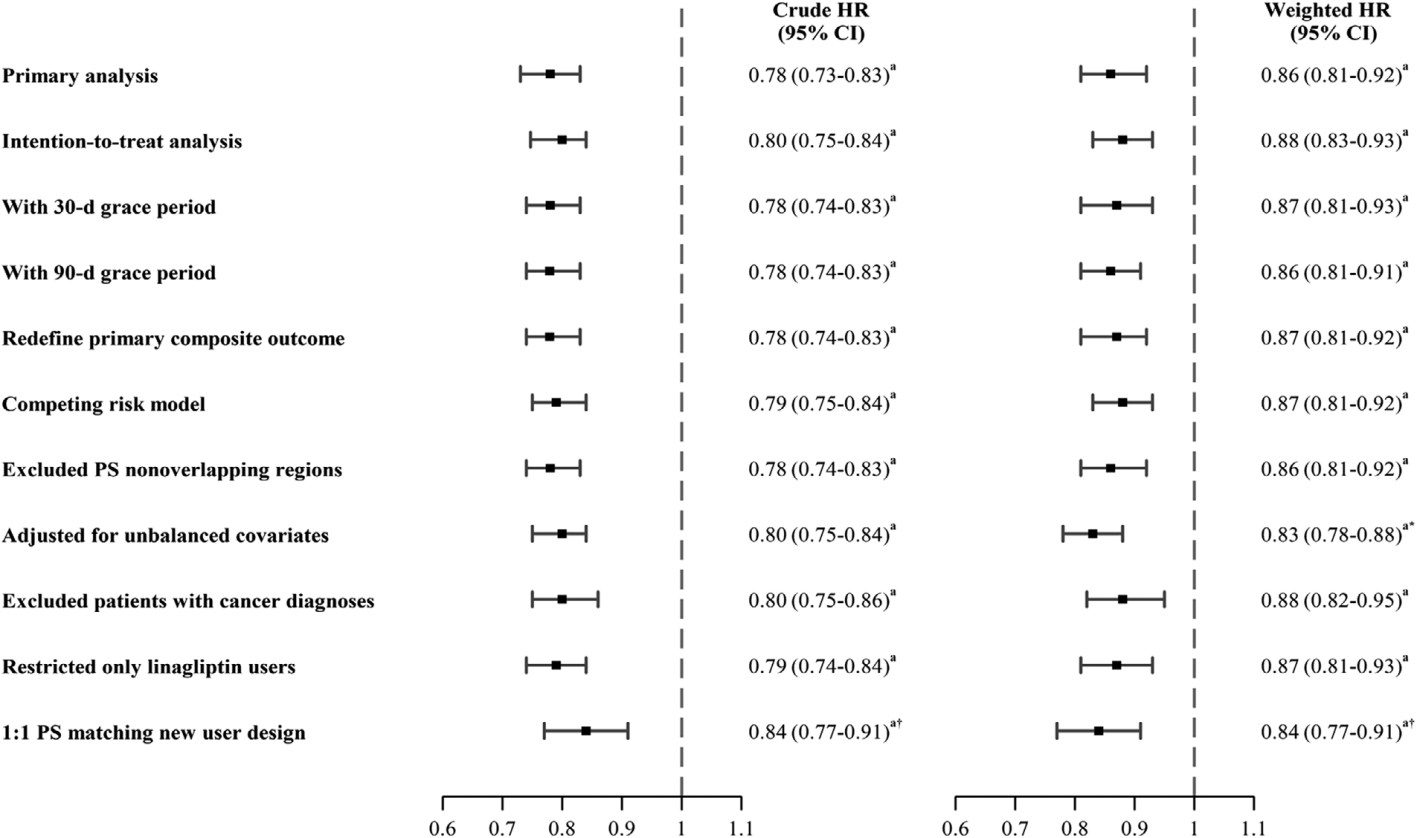
Forest plot of sensitivity analyses for the primary composite renal outcomes. CI, confidence interval; HR, hazard ratio; PS, propensity score. ^a^p < 0.05. ^*^HR with 95% CI was estimated by a multiple Cox regression model, which adjusted for number of test order for microalbuminuria and HbA1c, cohort entry year, monthly income‐based insurance premium, edema, antipsychotics, and use of ketosteril. †HR with 95% CI was estimated using a 1:1 PS matching approach and a new user design, which led to all balanced characteristics between the two groups.

**Fig. 2:**
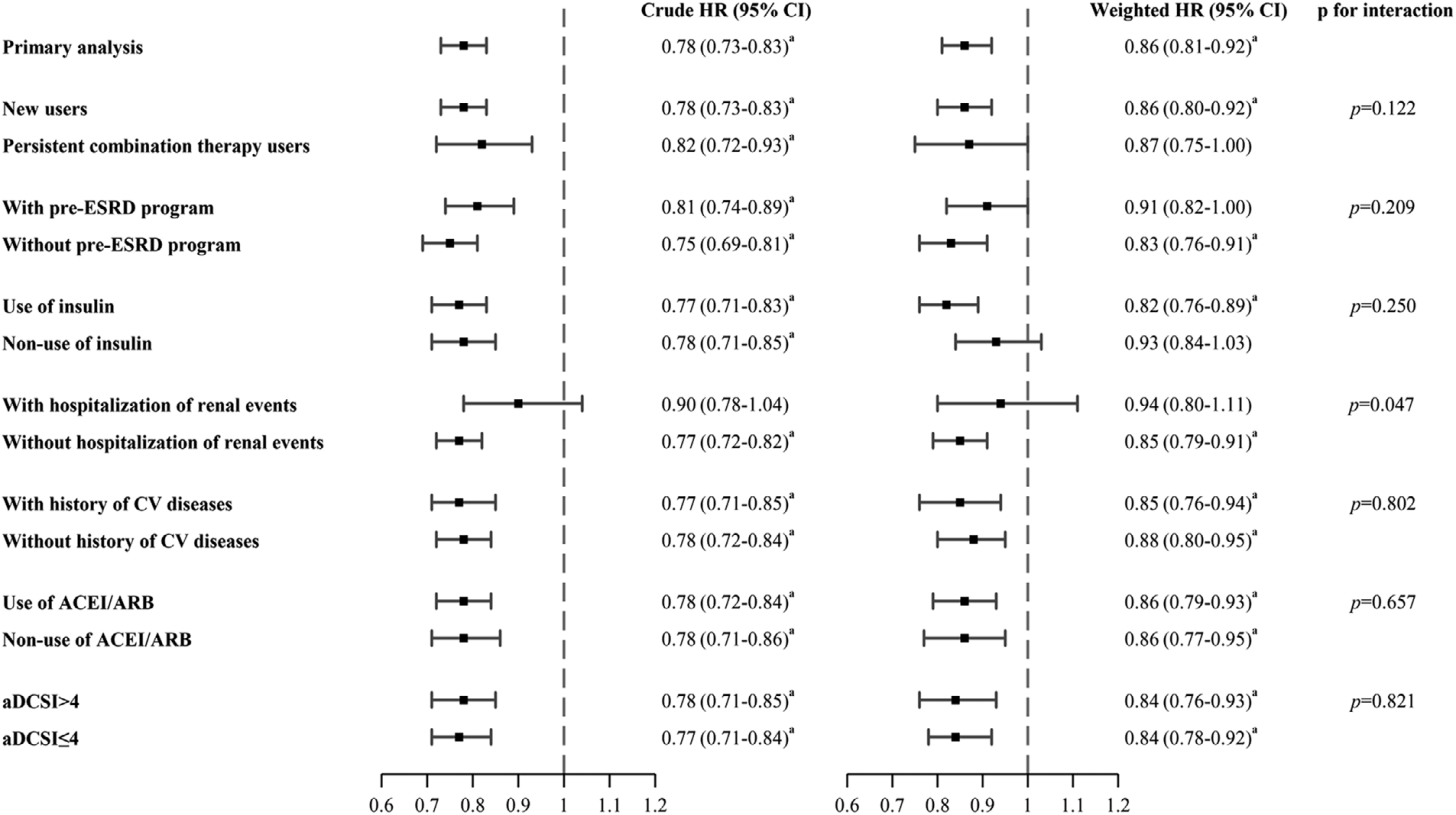
Forest plot of subgroup analyses for the primary composite renal outcomes. ACEIs, angiotensin‐converting enzyme inhibitors; aDCSI, adapted Diabetes Complication Index; ARB, angiotensin II receptor blockers; CI, confidence interval; CV, cardiovascular; ESRD, end‐stage renal disease; HR, hazard ratio. ^a^p < 0.05.

The primary findings remained consistent across all sensitivity analyses, including adopting 1‐year intention‐to‐treat analysis, treating non‐renal deaths as competing events, and restricting DPP‐4i users to linagliptin users. As expected, a null association was observed for the negative outcome (weighted HR, 1.00; 95% CI, 0.84–1.20) (Fig. 1 and Table S6). In addition, the estimated *E*‐value for the primary analysis was 1.6, indicating that our primary finding could be explained by potential unmeasured confounding if an unmeasured confounder is associated with both DPP‐4i use and renal composite outcome, with relative risks at least 1.6. No apparent significant effect modifiers were observed across the subgroup analyses, except for prior renal events, which had a *p*‐value of 0.047, approaching the threshold of 0.05 (Fig. 2 and Table S7).

## Discussion

This nationwide cohort study of pre‐dialysis patients with T2DM found that DPP‐4i were associated with a 14% lower risk of the composite outcome—comprising renal replacement therapy (dialysis or renal transplantation), renal deaths, or hospitalizations for kidney‐related events—compared with meglitinides. Further analysis of the composite outcome revealed that the decreased risk was primarily attributable to renal replacement therapy, showing a 17% reduction. DPP‐4i were also associated with a 41% reduced risk of hypoglycemia. Findings were consistent across all sensitivity analyses, supporting the robustness of the observed associations.

Empirical evidence on the renal effects of DPP‐4i in pre‐dialysis patients is limited. Previous studies evaluating DPP‐4i use and renal outcomes in patients with diabetes generally excluded those receiving dialysis or in pre‐dialysis stages. The CARMELINA trial enrolled patients with an eGFR of 15–90 mL/min/1.73 m^2^, and the SAVOR‐TIMI 53 trial included those with an eGFR of 30–90 mL/min/1.73 m^2^. Both trials reported reductions in albuminuria risk with linagliptin and saxagliptin, respectively; however, neither showed reduced risks of dialysis or renal transplantation [16, 17]. Additionally, two observational studies found that DPP‐4i use was associated with slower eGFR decline rates compared with nonuse, although potential confounding by indication bias may exist [44, 45]. Yang et al. reported no significant difference in the risk of renal replacement therapy between DPP‐4i and sulfonylureas among patients with diabetes and CKD stages 3b to 5 [46]. However, their findings were potentially limited by small sample sizes and reduced generalizability, as patients who received EPO—used by more than 60% of patients with CKD stage 5 [15, 22]—were excluded. Notably, pre‐dialysis patients were largely unrepresented across these studies, except for a small number of patients with CKD stage 5 in Yang's study. Therefore, the present study addresses a critical evidence gap and informs clinical decision‐making for this high‐risk population.

We observed that DPP‐4i may slow the progression to dialysis or renal transplantation in patients with T2DM and CKD stage 5, despite a short follow‐up period. The CARMELINA trial showed a reduction in albuminuria with linagliptin, which emerged right after approximately 6 months of therapy, although the median follow‐up was 2 years [16]. In contrast, our study found that a mean treatment duration of approximately 5 months with DPP‐4i was related to a lower risk of renal progression. However, whether short‐term DPP‐4i use can reduce the risk of albuminuria and, consequently, decrease the risk of progression to renal replacement therapy remains uncertain. Although real‐world studies suggest that DPP‐4i may slow down the decline in eGFR and progression to ESRD in patients with mild‐to‐moderate CKD [44, 45, 47], it is unclear whether these effects apply to those with advanced CKD. Accordingly, it remains unclear whether the reduced risk of progression to dialysis or renal transplantation with DPP‐4i in pre‐dialysis patients is mediated by a reduction in albuminuria or slower eGFR decline.

The observed protective effect of DPP‐4i on severe renal outcomes, including dialysis, may involve both GLP‐1 receptor (GLP‐1R)‐dependent [48] and GLP‐1R‐independent pathways [49]. DPP‐4i inhibits the breakdown of incretin hormones, mainly GLP‐1 and gastric inhibitory peptide, and their renal effects could be mediated through GLP‐1R‐dependent mechanisms, similar to those identified for GLP‐1 receptor agonists (GLP‐1 RA). Under chronic hyperglycemia, GLP‐1R signaling helps protect against renal oxidative stress by inhibiting NAD(P) oxidase, a major source of glomerular superoxide, and by activating the cyclic AMP‐protein kinase A pathway [48]. These effects may contribute to the renal protection associated with DPP‐4i. Additionally, GLP‐1R‐independent pathways also play a role in DPP‐4i‐associated renal protection effects, with studies suggesting that DPP‐4i influences signaling pathways beyond the GLP‐1/GLP‐1R pathway, involving heterogeneous nuclear ribonucleoprotein A, collagen I hemostasis, box‐binding protein‐1, thymosin β4, and renal transforming growth factor β1 [49].

To our knowledge, this is the first study assessing whether use of DPP‐4i versus meglitinides reduces the risk of progression to renal replacement therapy or renal‐specific mortality in pre‐dialysis patients with T2DM and CKD stage 5 not receiving dialysis. Given the similar pharmacotherapeutic roles of DPP‐4i and meglitinides in this population, baseline characteristics were largely balanced between the two groups before applying IPTW, indicating strong comparability. The use of meglitinides as an active‐comparator also helped mitigate confounding by indication. Both new and prevalent users were included to reflect real‐world prescribing patterns. The algorithms used to define Type 2 diabetes, CKD stage 5, and most outcomes have been validated with high accuracy [21, 23, 24]. The primary findings remained robust across multiple sensitivity analyses, supporting the robustness of our findings.

Our study has several limitations. First, not all patients with diabetes and CKD stage 5 were included. Patients with CKD stage 5 were identified based on prescription refill records for EPO, which are indicated for anemia and reimbursed for those with an eGFR less than 15 mL/min/1.73 m^2^. Although not all patients with CKD stage 5 receive EPO, anemia is prevalent in this population, and studies report that 60%–85% of these patients in Taiwan are treated with EPO [15, 22]. Second, we were unable to measure certain confounders, such as blood glucose levels, eGFR and albuminuria; therefore, unmeasured confounding could either overestimate or underestimate the observed association. However, we considered several proxy indicators of diabetes and CKD severity at baseline. Additionally, we adopted GERD as a negative control outcome and observed no association, suggesting minimum influence from unmeasured confounding. The estimated *E*‐value of 1.6 indicates that an unmeasured confounder would need to be 1.6 times more prevalent in the comparator group than in the DPP‐4i group and increase the risk of severe renal outcomes by a factor of 1.6 to fully account for the observed association. This suggests our results are unlikely fully attributable to unmeasured confounding. Third, the inclusion of prevalent users may introduce bias. However, we restricted prevalent users to those receiving a combination of DPP‐4i and meglitinides prior to starting EPO and ensured the duration of the combined treatment was similar between the two groups to minimize prevalent user bias. Accordingly, the impact of this bias is likely minimal, though it could lead to either overestimation or underestimation of the estimated HR. We also repeated the analysis using a new‐user design coupled with 1:1 PS matching, and the findings remained consistent. Fourth, the short duration of DPP‐4i and meglitinide use may reflect the rapid progression to renal replacement therapy or renal‐related hospitalization in patients with diabetes and severe renal disease, making it challenging to evaluate the long‐term renal effects of DPP‐4i. However, this short‐term usage reflects real‐world clinical practice. Lastly, our findings may be influenced by the ethnic composition of the study cohort. Yet, approximately 96% of Taiwanese inhabitants are Han Chinese [50], suggesting limited ethnic heterogeneity and minimal impact on the internal validity of our findings. As noted in the first limitation, the use of EPO as one of the inclusion criteria may limit the representativeness of the broader Taiwanese population with diabetes and CKD stage 5. Nevertheless, a study from a Taiwanese tertiary hospital using medical records to identify patients with diabetes and CKD stage 5 reported similar demographic and clinical characteristics to those in our study [51], supporting the generalizability of our findings to this population in Taiwan. Additionally, international evidence on individuals with diabetes and CKD stage 5 remains limited. For instance, recent studies from Australia and Pakistan, which included this vulnerable population, had small sample sizes, assessed few potential confounders, and did not evaluate renal disease progression, making comparisons with our study regarding patient characteristics challenging [52, 53]. These gaps highlight the novelty of our study and underscore the need for further research using real‐world data from diverse geographic and ethnic populations to validate our findings.

## Conclusions

This large nationwide cohort study found that the use of DPP‐4i was associated with a significantly decreased risk of composite renal events, including renal replacement, renal death, and hospitalization of kidney‐related events, compared to meglitinides in patients with T2DM and CKD stage 5 not on dialysis. Additionally, DPP‐4i use was linked to a reduced risk of hypoglycemia compared to meglitinides. Our findings suggest that, for pre‐dialysis patients with T2DM, DPP‐4 inhibitors may provide more renal advantages and less hypoglycemia risk than meglitinides.

## Author contributions

All authors conceptualized and contributed to the design of the current study. Meng‐Ting Wang acquired data from the database. Meng‐Ting Wang and Tung‐Ying Hung analyzed the data. Tung‐Ying Hung, Liang‐Yu Lin, Ying‐Jay Liou, Yu‐Juei Hsu, and Tzu‐Han Lin interpreted the data. Meng‐Ting Wang, Tung‐Ying Hung, and Tzu‐Chieh Lin drafted the manuscript. All authors thoroughly reviewed and approved the submitted manuscript.

## Disclosure

During the course of preparing this work, the authors used ChatGPT 4.0 to only edit the manuscript for enhancing manuscript readability and refining English expression. Following the use of this tool, the authors formally reviewed the content for its accuracy and edited it as necessary. The authors take full responsibility for all the content of this publication.

## Conflict of interest statement

All authors declare that there is no conflict of interest.

## Supporting information




**Table S1**. Operational definition of the inclusion and exclusion criteria, exposures, outcomes, comorbidities, and comedications.
**Table S2**. Schoenfeld residual tests for assessing proportionality hazard assumptions by outcomes.
**Table S3**. All baseline characteristics of DPP‐4 inhibitor users versus meglitinide users before and after IPTW.
**Table S4**. The mean duration and the reasons for truncation during follow‐up for DPP‐4 inhibitors and meglitinides, by outcomes.
**Table S5**. The patient‐based number needed to treat for comparative results of DPP‐4 inhibitor versus meglitinide.
**Table S6**. Sensitivity analyses of the primary outcome associated with the use of DPP‐4 inhibitors versus meglitinides.
**Table S7**. Comparison of the primary outcome between DPP‐4 inhibitors and meglitinides, stratified by pre‐determined baseline characteristics.
**Figure S1**. Flow chart of the eligible study population.
**Figure S2**. Weighted Kaplan–Meier survival curves of the primary outcome (A), renal replacement therapy (B), renal death (C), and hospitalization of kidney‐related events (D) between users of DPP‐4 inhibitors and meglitinide.
**Figure S3**. Weighted Kaplan–Meier survival curves of 3‐point MACE (A), hospitalization of heart failure (B), all‐cause mortality (C), and hypoglycemia (D) between users of DPP‐4 inhibitors and meglitinides.
**Methods**. Sample size calculation.

## Data Availability

This study primarily employed claims data obtained from the Health and Welfare Data Source Center, Ministry of Health and Welfare (HWDC, MOHW) in Taiwan. Based on the regulations of Taiwan's Ministry of Health and Welfare (https://dep.mohw.gov.tw/DOS/cp‐5119‐59201‐113.html), public sharing of the claims data is prohibited. Please contact the HWDC, MOHW to request access to the analyzed data.

## References

[1] Hoogeveen EK . The epidemiology of diabetic kidney disease. Kidney Dial. 2022;2:433–42.

[2] International Diabetes Federation . IDF diabetes atlas. 10th ed. Brussels: International Diabetes Federation; 2021.

[3] International Diabetes Federation . Diabetes atlas reports: Diabetes and kidney disease. Brussels: International Diabetes Federation; 2023.

[4] Murtagh FE , Sheerin NS , Addington‐Hall J , Higginson IJ . Trajectories of illness in stage 5 chronic kidney disease: A longitudinal study of patient symptoms and concerns in the last year of life. Clin J Am Soc Nephrol. 2011;6:1580–90. 21685021 10.2215/CJN.09021010

[5] Fletcher BR , Damery S , Aiyegbusi OL , Anderson N , Calvert M , Cockwell P , et al. Symptom burden and health‐related quality of life in chronic kidney disease: A global systematic review and meta‐analysis. PLoS Med. 2022;19:e1003954. 35385471 10.1371/journal.pmed.1003954 PMC8985967

[6] Deng Y , Li N , Wu Y , Wang M , Yang S , Zheng Y , et al. Global, regional, and national burden of diabetes‐related chronic kidney disease from 1990 to 2019. Front Endocrinol (Lausanne). 2021;12:672350. 34276558 10.3389/fendo.2021.672350 PMC8281340

[7] Bello AK , Okpechi IG , Levin A , Ye F , Damster S , Arruebo S , et al. An update on the global disparities in kidney disease burden and care across world countries and regions. Lancet Glob Health. 2024;12:e382–e95. 38365413 10.1016/S2214-109X(23)00570-3

[8] Perkovic V , Jardine MJ , Neal B , Bompoint S , Heerspink HJL , Charytan DM , et al. Canagliflozin and renal outcomes in type 2 diabetes and nephropathy. N Engl J Med. 2019;380:2295–306. 30990260 10.1056/NEJMoa1811744

[9] Herrington WG , Staplin N , Wanner C , Green JB , Hauske SJ , Emberson JR , et al. Empagliflozin in patients with chronic kidney disease. N Engl J Med. 2023;388:117–27. 36331190 10.1056/NEJMoa2204233 PMC7614055

[10] Heerspink HJL , Stefánsson BV , Correa‐Rotter R , Chertow GM , Greene T , Hou F‐F , et al. Dapagliflozin in patients with chronic kidney disease. N Engl J Med. 2020;383:1436–46. 32970396 10.1056/NEJMoa2024816

[11] Rossing P , Caramori ML , Chan JCN , Heerspink HJL , Hurst C , Khunti K , et al. KDIGO 2022 Clinical practice guideline for diabetes management in chronic kidney disease. Kidney Int. 2022;102:S1–S127. 36272764 10.1016/j.kint.2022.06.008

[12] Badwan OZ , Braghieri L , Skoza W , Agrawal A , Menon V , Tang WHW . When should we consider SGLT‐2 inhibitors in patients with acute decompensated heart failure? Cleve Clin J Med. 2024;91:47–51. 38167393 10.3949/ccjm.91a.23034

[13] Mann JFE , Ørsted DD , Brown‐Frandsen K , Marso SP , Poulter NR , Rasmussen S , et al. Liraglutide and renal outcomes in type 2 diabetes. N Engl J Med. 2017;377:839–48. 28854085 10.1056/NEJMoa1616011

[14] Perkovic V , Tuttle KR , Rossing P , Mahaffey KW , Mann JFE , Bakris G , et al. Effects of semaglutide on chronic kidney disease in patients with type 2 diabetes. N Engl J Med. 2024;391:109–121. 38785209 10.1056/NEJMoa2403347

[15] National Health Research Institutes & Taiwan Society of Nephrology . 2022 annual report on kidney disease in Taiwan [Electronic book, in Traditional Chinese]. Zhunan and Taipei, Taiwan: National Health Research Institutes and Taiwan Society of Nephrology. 2023; Retrieved June 30, 2025, from https://www.tsn.org.tw/twrds_detail.html?year=2022

[16] Rosenstock J , Perkovic V , Johansen OE , Cooper ME , Kahn SE , Marx N , et al. Effect of linagliptin vs placebo on major cardiovascular events in adults with type 2 diabetes and high cardiovascular and renal risk: The carmelina randomized clinical trial. JAMA. 2019;321:69–79. 30418475 10.1001/jama.2018.18269 PMC6583576

[17] Mosenzon O , Leibowitz G , Bhatt DL , Cahn A , Hirshberg B , Wei C , et al. Effect of saxagliptin on renal outcomes in the SAVOR‐TIMI 53 trial. Diabetes Care. 2017;40:69–76. 27797925 10.2337/dc16-0621

[18] Bae JH , Kim S , Park EG , Kim SG , Hahn S , Kim NH . Effects of dipeptidyl peptidase‐4 inhibitors on renal outcomes in patients with type 2 diabetes: A systematic review and meta‐analysis. Endocrinol Metab (Seoul). 2019;34:80–92. 30912341 10.3803/EnM.2019.34.1.80 PMC6435854

[19] Lin LY , Warren‐Gash C , Smeeth L , Chen PC . Data resource profile: The National Health Insurance Research Database (NHIRD). Epidemiol Health. 2018;40:e2018062. 30727703 10.4178/epih.e2018062 PMC6367203

[20] Huang YT , Wei T , Huang YL , Wu YP , Chan KA . Validation of diagnosis codes in healthcare databases in Taiwan, a literature review. Pharmacoepidemiol Drug Saf. 2023;32:795–811. 36890603 10.1002/pds.5608

[21] Chen H‐Y , Sun C‐Y , Lee C‐C , Wu I‐W , Chen Y‐C , Lin Y‐H , et al. Ketoanalogue supplements reduce mortality in patients with pre‐dialysis advanced diabetic kidney disease: A nationwide population‐based study. Clin Nutr. 2021;40:4149–60. 33597108 10.1016/j.clnu.2021.01.045

[22] Hsu T‐W , Liu J‐S , Hung S‐C , Kuo K‐L , Chang Y‐K , Chen Y‐C , et al. Renoprotective effect of renin‐angiotensin‐aldosterone system blockade in patients with predialysis advanced chronic kidney disease, hypertension, and anemia. JAMA Intern Med. 2014;174:347–54. 24343093 10.1001/jamainternmed.2013.12700

[23] Sung SF , Hsieh CY , Lin HJ , Chen YW , Yang YH , Li CY . Validation of algorithms to identify stroke risk factors in patients with acute ischemic stroke, transient ischemic attack, or intracerebral hemorrhage in an administrative claims database. Int J Cardiol. 2016;215:277–82. 27128546 10.1016/j.ijcard.2016.04.069

[24] Hsieh MT , Hsieh CY , Tsai TT , Sung SF . Validation of stroke risk factors in patients with acute ischemic stroke, transient ischemic attack, or intracerebral hemorrhage on Taiwan's National Health Insurance claims data. Clin Epidemiol. 2022;14:327–35. 35330593 10.2147/CLEP.S353435 PMC8938165

[25] Scott LJ . Repaglinide: A review of its use in type 2 diabetes mellitus. Drugs. 2012;72:249–72. 22268393 10.2165/11207600-000000000-00000

[26] Hasslacher C . Safety and efficacy of repaglinide in type 2 diabetic patients with and without impaired renal function. Diabetes Care. 2003;26:886–91. 12610054 10.2337/diacare.26.3.886

[27] Pasternak B , Wintzell V , Melbye M , Eliasson B , Svensson A‐M , Franzén S , et al. Use of sodium‐glucose co‐transporter 2 inhibitors and risk of serious renal events: Scandinavian cohort study. BMJ. 2020;369:m1186. 32349963 10.1136/bmj.m1186 PMC7188014

[28] Pasternak B , Wintzell V , Eliasson B , Svensson A‐M , Franzén S , Gudbjörnsdottir S , et al. Use of glucagon‐like peptide 1 receptor agonists and risk of serious renal events: Scandinavian cohort study. Diabetes Care. 2020;43:1326–35. 32295809 10.2337/dc19-2088

[29] Cheng CL , Lee CH , Chen PS , Li YH , Lin SJ , Yang YH . Validation of acute myocardial infarction cases in the national health insurance research database in Taiwan. J Epidemiol. 2014;24:500–7. 25174915 10.2188/jea.JE20140076 PMC4213225

[30] Hsieh CY , Chen CH , Li CY , Lai ML . Validating the diagnosis of acute ischemic stroke in a National Health Insurance claims database. J Formos Med Assoc. 2015;114:254–9. 24140108 10.1016/j.jfma.2013.09.009

[31] Hsieh MT , Hsieh CY , Tsai TT , Wang YC , Sung SF . Performance of ICD‐10‐CM Diagnosis Codes for identifying acute ischemic stroke in a National Health Insurance claims database. Clin Epidemiol. 2020;12:1007–13. 33061648 10.2147/CLEP.S273853 PMC7524174

[32] Lin Y‐S , Chen T‐H , Chi C‐C , Lin M‐S , Tung T‐H , Liu C‐H , et al. Different implications of heart failure, ischemic stroke, and mortality between nonvalvular atrial fibrillation and atrial flutter—a view from a National Cohort Study. J Am Heart Assoc. 2017;6: e006406. 28733435 10.1161/JAHA.117.006406 PMC5586326

[33] Ginde AA , Blanc PG , Lieberman RM , Camargo CA Jr . Validation of ICD‐9‐CM coding algorithm for improved identification of hypoglycemia visits. BMC Endocr Disord. 2008;8:4. 18380903 10.1186/1472-6823-8-4 PMC2323001

[34] Karter AJ , Warton EM , Moffet HH , Ralston JD , Huang ES , Miller DR , et al. Revalidation of the hypoglycemia risk stratification tool using ICD‐10 codes. Diabetes Care. 2019;42:e58–e9. 30765427 10.2337/dc18-2154 PMC6429629

[35] Chang HY , Weiner JP , Richards TM , Bleich SN , Segal JB . Validating the adapted Diabetes Complications Severity Index in claims data. Am J Manag Care. 2012;18:721–6. 23198714

[36] Fine JP , Gray RJ . A proportional hazards model for the subdistribution of a competing risk. J Am Stat Assoc. 1999;94:496–509.

[37] Noguchi Y , Katsuno H , Ueno A , Otsubo M , Yoshida A , Kanematsu Y , et al. Signals of gastroesophageal reflux disease caused by incretin‐based drugs: A disproportionality analysis using the Japanese adverse drug event report database. J Pharm Health Care Sci. 2018;4:15. 29946474 10.1186/s40780-018-0109-z PMC6004661

[38] Ioannidis JPA , Tan YJ , Blum MR . Limitations and Misinterpretations of E‐values for sensitivity analyses of observational studies. Ann Intern Med. 2019;170:108–11. 30597486 10.7326/M18-2159

[39] Chesnaye NC , Stel VS , Tripepi G , Dekker FW , Fu EL , Zoccali C , et al. An introduction to inverse probability of treatment weighting in observational research. Clin Kidney J. 2022;15:14–20. 35035932 10.1093/ckj/sfab158 PMC8757413

[40] Austin PC . An introduction to propensity score methods for reducing the effects of confounding in observational studies. Multivariate Behav Res. 2011;46:399–424. 21818162 10.1080/00273171.2011.568786 PMC3144483

[41] Xu S , Ross C , Raebel MA , Shetterly S , Blanchette C , Smith D . Use of stabilized inverse propensity scores as weights to directly estimate relative risk and its confidence intervals. Value Health. 2010;13:273–7. 19912596 10.1111/j.1524-4733.2009.00671.x PMC4351790

[42] Abeysekera WWM , Sooriyarachchi R . Use of Schoenfeld's global test to test the proportional hazards assumption in the Cox proportional hazards model: An application to a clinical study. J Natl Sci Found Sri Lanka. 2009;37:41–51.

[43] Suissa D , Brassard P , Smiechowski B , Suissa S . Number needed to treat is incorrect without proper time‐related considerations. J Clin Epidemiol. 2012;65:42–6. 21816576 10.1016/j.jclinepi.2011.04.009

[44] Esaki H , Tachi T , Goto C , Sugita I , Kanematsu Y , Yoshida A , et al. Renoprotective effect of dipeptidyl peptidase‐4 inhibitors in patients with type 2 diabetes mellitus. Front Pharmacol. 2017;8:835. 29187821 10.3389/fphar.2017.00835 PMC5694778

[45] Hsu WC , Lin CS , Chen JF , Chang CM . The effects of dipeptidyl peptidase 4 inhibitors on renal function in patients with type 2 diabetes mellitus. J Clin Med. 2022;11:2653. 35566779 10.3390/jcm11092653 PMC9101888

[46] Yang CT , Lin WH , Li LJ , Ou HT , Kuo S . Association of renal and cardiovascular safety with DPP‐4 inhibitors vs. sulfonylureas in patients with type 2 diabetes and advanced chronic kidney disease. Clin Pharmacol Ther. 2021;110:464–72. 33866549 10.1002/cpt.2262

[47] Hashimoto H , Satoh M , Nakayama S , Toyama M , Murakami T , Obara T , et al. Comparison of renal prognosis between dipeptidyl peptidase‐4 inhibitor users and non‐users. Diabetes, Obes Metabo. 2024;26:4460–7. 10.1111/dom.15800 39086031

[48] Fujita H , Morii T , Fujishima H , Sato T , Shimizu T , Hosoba M , et al. The protective roles of GLP‐1R signaling in diabetic nephropathy: Possible mechanism and therapeutic potential. Kidney Int. 2014;85:579–89. 24152968 10.1038/ki.2013.427

[49] Hasan AA , von Websky K , Reichetzeder C , Tsuprykov O , Gaballa MMS , Guo J , et al. Mechanisms of GLP‐1 receptor–independent renoprotective effects of the dipeptidyl peptidase type 4 inhibitor linagliptin in GLP‐1 receptor knockout mice with 5/6 nephrectomy. Kidney Int. 2019;95:1373–88. 30979564 10.1016/j.kint.2019.01.010

[50] Le Pesant T . Generational change and ethnicity among 1980s‐born Taiwanese. J Curr Chin Aff. 2011;40:133–57.

[51] Kuo I‐C , Lin HY‐H , Niu S‐W , Hwang D‐Y , Lee J‐J , Tsai J‐C , et al. Glycated hemoglobin and outcomes in patients with advanced diabetic chronic kidney disease. Sci Rep. 2016;6:20028. 26818011 10.1038/srep20028 PMC4730215

[52] Vankwani R , Kumar M , Mal P , Gurbukshani S . Association between microvascular complications and chronic kidney disease stages in type 2 diabetic patients. J Shalamar Medical & Dental College. 2024;5:71–6.

[53] Lunardi LE , K Le Leu R , Matricciani LA , Xu Q , Britton A , Jesudason S , et al. Patient activation in advanced chronic kidney disease: A cross‐sectional study. J Nephrol. 2024;37:343–52. 38345687 10.1007/s40620-023-01847-x PMC11043190

