# Supplementary material for: Use of dipeptidyl peptidase‐4 inhibitors is associated with lower risk of severe renal outcomes in pre‐dialysis patients with Type 2 diabetes
Source: J Intern Med. 2025 Jul 3;298(3):214–27. doi: 10.1111/joim.20112 (PMC12374763; doi:10.1111/joim.20112)
Supplement: Supplementary file 1 — Table S1. Operational definition of the inclusion and exclusion criteria, exposures, outcomes, comorbidities, and comedications. Table S2. Schoenfeld residual tests for assessing proportionality hazard assumptions by outcomes. Table S3. All baseline characteristics of DPP‐4 inhibitor users versus meglitinide users before and after IPTW. Table S4. The mean duration and the reasons for truncation during follow‐up for DPP‐4 inhibitors and meglitinides, by outcomes. Table S5. The patient‐based number needed to treat for comparative results of DPP‐4 inhibitor versus meglitinide. Table S6. Sensitivity analyses of the primary outcome associated with the use of DPP‐4 inhibitors versus meglitinides. Table S7. Comparison of the primary outcome between DPP‐4 inhibitors and meglitinides, stratified by pre‐determined baseline characteristics. Figure S1. Flow chart of the eligible study population. Figure S2. Weighted Kaplan–Meier survival curves of the primary outcome (A), renal replacement therapy (B), renal death (C), and hospitalization of kidney‐related events (D) between users of DPP‐4 inhibitors and meglitinide. Figure S3. Weighted Kaplan–Meier survival curves of 3‐point MACE (A), hospitalization of heart failure (B), all‐cause mortality (C), and hypoglycemia (D) between users of DPP‐4 inhibitors and meglitinides. Methods. Sample size calculation. [file JOIM-298-214-s001.docx]

**Supplementary files for “Use of dipeptidyl peptidase-4 inhibitors is associated with lower risk of severe renal outcomes in pre-dialysis patients with type 2 diabetes"**

**eTable 1. Operational definition of the inclusion and exclusion criteria, exposures, outcomes, comorbidities, and comedications**

**eTable 2. Schoenfeld residuals tests for assessing proportionality hazard assumptions by outcomes**

**eTable 3. All baseline characteristics of DPP-4 inhibitor users versus meglitinide users before and after IPTW**

**eTable 4. The mean duration and the reasons for truncation during follow-up for DPP-4 inhibitors and meglitinides, by outcomes**

**eTable 5. The patient-based number needed to treat for comparative results of DPP-4 inhibitor versus meglitinide**

**eTable 6. Sensitivity analyses of the primary outcome associated with the use of DPP-4 inhibitors versus meglitinides**

**eTable 7. Comparison of the primary outcome between DPP-4 inhibitors and meglitinides, stratified by pre-determined baseline characteristics**

**eFigure 1. Flow chart of the eligible study population.**

**eFigure 2. Weighted Kaplan Meier survival curves of the primary outcome (A), renal replacement therapy (B), renal death (C) and hospitalization of kidney-related events (D) between users of DPP-4 inhibitors and meglitinide.**

**eFigure 3. Weighted Kaplan Meier survival curves of 3-point MACE (A), hospitalization of heart failure (B), all-cause mortality (C), hypoglycemia (D) between users of DPP-4 inhibitors and meglitinides.**

eMethods. Sample size calculation

eTable 1. Operational definition of the inclusion and exclusion criteria, exposures, outcomes, comorbidities, and comedications

| **Inclusion criteria** | **Operational definition** |
| --- | --- |
| Diabetes | ICD-9-CM codes 250; ICD-10-CM codes E10-E14 |
| Stage 5 CKD | Received an erythropoietin-stimulating agent (EPO), including epoetin alfa, methoxy polyethylene glycol-epoetin beta, epoetin beta, recombinant human erythropoietin and darbepoetin alfa |
| **Exclusion criteria** | **Operational definition** |
| Renal replacement therapy | Catastrophic illness file: ICD-9-CM codes 585, 403.01, 403.11, 403.91, 404.02, 404.03, 404.12, 404.13, 404.92, 404.93；ICD-10-CM codes N18.5, N18.6, I12.0, I13.11, I13.2。 |
| Type 1 diabetes | ICD-9-CM codes 250.x1, 250.x3; ICD-10-CM codes E11 |
| Gestational diabetes | ICD-9-CM codes 648.8; ICD-10-CM codes O24.4 |
| **Exposures** | **Individual drugs** |
| Meglitinides | Mitiglinide, nateglinide, and repaglinide |
| Dipeptidyl peptidase-4 inhibitors | Alogliptin, linagliptin, saxagliptin, sitagliptin and vildagliptin |
| **Outcome definitions** | **Operational definition** |
| Composite renal outcome | Including renal replacement (dialysis or kidney transplantation), renal death, and hospitalization for kidney-related events, detail in below. |
| Renal replacement therapy | Catastrophic illness file: ICD-9-CM codes 585, 403.01, 403.11, 403.91, 404.02, 404.03, 404.12, 404.13, 404.92, 404.93；ICD-10-CM codes N18.5, N18.6, I12.0, I13.11, I13.2。 |
| Renal death | Nationwide death registry file: ICD-10-CM codes: E11.2, E13.2, E14.2, I12.0, I13.1, I13.2, N00-N08, N10-N23, N25-N29 |
| Hospitalization for kidney-related events | ICD-9-CM codes 250.40, 250.42, 403.01, 403.11, 403.91, 404.02, 404.03, 404.12, 404.13, 404.92, 404.93, 583.6, 583.7, 583.81, 584.5-584.9, 585, 586  ICD-10-CM codes E11.2, E13.2, I12.0, I13.1, I13.2, N17-N19 |
| 3-point major adverse cardiovascular events (3P-MACE) | Myocardial infraction: ICD-9-CM codes 410; ICD-10-CM codes I21  Ischemic stroke: ICD-9-CM codes 433-434; ICD-10-CM codes I63  Cardiovascular death: Nationwide death registry file ICD-10-CM codes I00-I99 |
| Hospitalization for heart failure | ICD-9-CM codes 428; ICD-10-CM codes I50 |
| Hypoglycemia | ICD-9-CM codes 251.0, 251.1, 251.2, 962.3, 250.8 (250.8 accompanied by following codes were excluded: 259.8, 272.7, 681, 682, 686.9, 707, 709.3, 730.0, 730.1, 730.2, 731.8); ICD-10-CM codes E08.641, E08.649, E09.641, E09.649, E11.641, E11.649, E13.641, E13.649, E15, E16.0, E16.1, E16.2, T38.3 (T38.3 accompanied by following codes were excluded: T38.3X6) |
| **Healthcare utilization** | **Operational definition** |
| Serum creatinine tests | NHI procedure codes 09015C |
| Microalbuminuria tests | NHI procedure codes 12111C, 27065B |
| HbA1c test | NHI procedure codes 09006C |
| Lipid tests | NHI procedure codes 09001C, 09001CA, 09004C, 09044C, 09043C |
| **Proxy indicators** | **Operational definition** |
| Hypoglycemia | ICD-9-CM codes 251.0, 251.1, 251.2, 962.3, 250.8 (250.8 accompanied by following codes were excluded: 259.8, 272.7, 681, 682, 686.9, 707, 709.3, 730.0, 730.1, 730.2, 731.8); ICD-10-CM codes E08.641, E08.649, E09.641, E09.649, E11.641, E11.649, E13.641, E13.649, E15, E16.0, E16.1, E16.2, T38.3 (T38.3 accompanied by following codes were excluded: T38.3X6) |
| Acute kidney disease | ICD-9-CM codes 584; ICD-10-CM codes N17 |
| Proteinuria | ICD-9-CM codes 791.0; ICD-10-CM codes R80.0, R80.1, R80.3, R80.8, R80.9 |
| Disorders of electrolyte | ICD-9-CM codes 276.0, 276.1, 276.7, 276.8, 276.9; ICD-10-CM codes E87.0, E87.1, E87.5, E876, E87.8 |
| Disorders of fluid balance | ICD-9-CM codes 276.5, 276.6; ICD-10-CM codes E86.0, E86.1, E86.9, E87.70 |
| Edema | ICD-9-CM codes 782.3; ICD-10-CM codes R60.0, R60.1 |
| Kidney and urinary stone | ICD-9-CM codes 592.0, 592.1, 592.9; ICD-10-CM codes N22, N20.0, N20.1, N20.2, N20.9 |
| **Comorbidities** | **Operational definition** |
| Myocardial infraction | ICD-9-CM codes 410; ICD-10-CM codes I21 |
| Ischemic stroke | ICD-9-CM codes 433-434; ICD-10-CM codes I63 |
| Hemorrhage stroke | ICD-9-CM codes 430-432; ICD-10-CM codes I60, I61 |
| Other stroke | ICD-9: 435-438; ICD-10: I62, I67-I69, G45-46 |
| Heart failure | ICD-9-CM codes 428; ICD-10-CM codes I50 |
| Peripheral arterial disease | ICD-9-CM codes 440, 443.9; ICD-10-CM codes I70, I75, I73.9 |
| Venous thromboembolism | ICD-9-CM codes 451-453, 415.1; ICD-10-CM codes I80-I82 |
| Coronary revascularization | ICD-9-PCS codes 00.6 6, 17.55, 36.0, 36.1, 36.2; ICD-10-PCS codes 0210093, 0210098, 0210099, 021009C, 021009F, 021009W, 02100A3, 02100A8, 02100A9, 02100AC, 02100AF, 02100AW, 02100J3, 02100J8, 02100J9, 02100JC, 02100JF, 02100JW, 02100K3, 02100K8, 02100K9, 02100KC, 02100KF, 02100KW, 02100Z3, 02100Z8, 02100Z9, 02100ZC, 02100ZF, 0210493, 0210498, 0210499, 021049C, 021049F, 021049W, 02104A3, 02104A8, 02104A9, 02104AC, 02104AF, 02104AW, 02104J3, 02104J8, 02104J9, 02104JC, 02104JF, 02104JW, 02104K3, 02104K8, 02104K9, 02104KC, 02104KF, 02104KW, 02104Z3, 02104Z8, 02104Z9, 02104ZC, 02104ZF, 0211093, 0211098, 0211099, 021109C, 021109F, 021109W, 02110A3, 02110A8, 02110A9, 02110AC, 02110AF, 02110AW, 02110J3, 02110J8, 02110J9, 02110JC, 02110JF, 02110JW, 02110K3, 02110K8, 02110K9,02110KC, 02110KF, 02110KW, 02110Z3, 02110Z8, 02110Z9, 02110ZC, 02110ZF, 0211493, 0211498, 0211499, 021149C, 021149F, 021149W, 02114A3, 02114A8, 02114A9, 02114AC, 02114AF, 02114AW, 02114J3, 02114J8, 02114J9, 02114JC, 02114JF, 02114JW, 02114K3, 02114K8, 02114K9, 02114KC, 02114KF, 02114KW, 02114Z3, 02114Z8, 02114Z9, 02114ZC, 02114ZF, 0212093, 0212098, 0212099, 021209C, 021209F, 021209W, 02120A3, 02120A8, 02120A9, 02120AC, 02120AF, 02120AW, 02120J3, 02120J8, 02120J9, 02120JC, 02120JF, 02120JW, 02120K3, 02120K8, 02120K9, 02120KC, 02120KF, 02120KW, 02120Z3, 02120Z8, 02120Z9, 02120ZC, 02120ZF, 0212493, 0212498, 0212499, 021249C, 021249F, 021249W, 02124A3, 02124A8, 02124A9, 02124AC, 02124AF, 02124AW, 02124J3, 02124J8, 02124J9, 02124JC, 02124JF, 02124JW, 02124K3, 02124K8, 02124K9, 02124KC, 02124KF, 02124KW, 02124Z3, 02124Z8, 02124Z9, 02124ZC, 02124ZF, 0213093, 0213098, 0213099, 021309C, 021309F, 021309W, 02130A3, 02130A8, 02130A9, 02130AC, 02130AF, 02130AW, 02130J3, 02130J8, 02130J9, 02130JC, 02130JF, 02130JW, 02130K3, 02130K8, 02130K9, 02130KC, 02130KF, 02130KW, 02130Z3, 02130Z8, 02130Z9, 02130ZC, 02130ZF, 0213493, 0213498, 0213499, 021349C, 021349F, 021349W, 02134A3, 02134A8, 02134A9, 02134AC, 02134AF, 02134AW, 02134J3, 02134J8, 02134J9, 02134JC, 02134JF, 02134JW, 02134K3, 02134K8, 02134K9, 02134KC, 02134KF, 02134KW, 02134Z3, 02134Z8, 02134Z9, 02134ZC, 02134ZF, 021K0Z8, 021K0Z9, 021K0ZC, 021K0ZF, 021K0ZW, 021K4Z8, 021K4Z9, 021K4ZC, 021K4ZF, 021K4ZW, 021L09P, 021L09Q, 021L09R, 021L0AP, 021L0AQ, 021L0AR, 021L0JP, 021L0JQ, 021L0JR, 021L0KP, 021L0KQ, 021L0KR, 021L0Z8或NHI procedure codes 68023B, 68024B, 68025B, 68053B, 68054B, 68055B, 3076B, 33077B, 33078B, N26002, N26003 |
| Hypertension | ICD-9-CM codes 401-405; ICD-10-CM codes I10-I15 |
| Dyslipidemia | ICD-9-CM codes 272; ICD-10-CM codes E71.30, E75.21, E75.22, E75.24, E75.3, E75.5, E75.6, E77, E78, E88.1, E88.2, E88.89 |
| Asthma | ICD-9-CM codes 493; ICD-10-CM codes J45 |
| Chronic obstructive pulmonary disease | ICD-9-CM codes 491, 492, 496; ICD-10-CM codes J41, J42, J43, J44 |
| Pneumonia | ICD-9-CM codes 480-486; ICD-10-CM codes J12-J18 |
| Depression | ICD-9-CM codes 296.2, 296.3, 300.4, 311; ICD-10-CM codes F32-33, F34.1 |
| Anxiety | ICD-9-CM codes 300; ICD-10-CM codes F40, F41 |
| Schizophrenia | ICD-9-CM codes 295; ICD-10-CM codes F20, F25 |
| Dementia | ICD-9-CM codes 290, 331; ICD-10-CM codes G30, F00, F01, F03, F05.1, G31.1, G31.82, G31.9 |
| Epilepsy | ICD-9-CM codes 345; ICD-10-CM codes G40 |
| Fracture | ICD-9-CM codes 800-829; ICD-10-CM codes S12, S22, S32, S42, S52, S62, S72, S82, S92, T02, T08, T10, T12 |
| Osteoporosis | ICD-9-CM codes 733.0; ICD-10-CM codes M81.0 |
| Osteoarthritis | ICD-9-CM codes 715; ICD-10-CM codes M15-M19 |
| Anemia | ICD-9-CM codes 280-285; ICD-10-CM codes D46.1, D46.4, D50-D64 |
| Thyroid disease | ICD-9-CM codes 240-246; ICD-10-CM codes E00-E07, E35, E89.0 |
| Liver disease | ICD-9-CM codes 571; ICD-10-CM codes K70-K76 |
| GERD | ICD 9: 530.11, 530.81; ICD 10: K21 |
| Autoimmune diseases | ICD-9-CM codes 099.3, 135, 136.1, 255.4, 287.31, 335.2, 340, 358.0, 374.53, 379.0, 390-392, 393-398, 416, 446.0, 446.4-5, 555, 556.9, 576.1, 579.0, 695.2, 695.4, 696.0-1, 701.0; ICD-10-CM codes M02.30, D086, M35.2, E27.1-E27.6, E89.6, D69.3, D69.4, G12.2, G12.8, G35, G70.0, H02.73, H15.0, H15.1, I00-I02, I05-I09, I27, M30, M31.3, M31.5-M31.7, K50, K51.9, K83.0, K90.0, L52, L93, L40, L90.0, L94.0, L94.1, L94.3 |
| Tobacco | ICD-9-CM codes 305.1; ICD-10-CM codes F17.2 |
| Obesity or weight gain | ICD-9-CM codes 278, V85.3, V85.4; ICD-10-CM codes E65-E68 |
| Alcohol-related disorder | ICD-9-CM codes 291, 303, 305.0; ICD-10-CM codes F10 |
| Acquired Immunodeficiency Syndrome | ICD-9-CM codes 042; ICD-10-CM codes M02.30, D086, M35.2, E27.1-E27.6, E89.6, D69.3, D69.4, G12.2, G12.8, G35, G70.0, H02.73, H15.0, H15.1, I00-I02, I05-I09, I27, M30, M31.3, M31.5-M31.7, K50, K51.9, K83.0, K90.0, L52, L93, L40, L90.0, L94.0, L94.1, L94.3 |
| **DM medication and comedications** | **Individual drugs** |
| Thiazolidinediones (TZD) | Pioglitazone and rosiglitazone |
| Glucagon-like peptide-1 receptor agonist (GLP-1RA) | Exenatide, Lixisenatide, Dulaglutide, Liraglutide and Semaglutide |
| Insulin | Insulin aspart, insulin glulisine, insulin human regular, insulin lispro, Insulin human NPH,  Insulin aspart 70/30, insulin lispro 50/50, insulin lispro 75/25, insulinNPH/regular 70/30, Insulin degludec, insulin detemir and insulin glargine |
| Biguanides | Buformin and metformin |
| Alpha-glucosidase inhibitors (AGI) | Acarbose and miglitol |
| Sodium glucose co-transport-2 inhibitors (SGLT2i) | Canagliflozin, dapagliflozin, empagliflozin and ertugliflozin |
| Angiotensin-converting enzyme inhibitor (ACEIs) | Benazepril, captopril, cilazapril, enalapril, fosinopril, imidapril, lisinopril, perindopril, quinapril and ramipril |
| Angiotensin receptor blockers (ARBs) | Azilsartan, candesartan, eprosartan, irbesartan, losartan, olmesartan, telmisartan and valsartan |
| α-agonists | Methyldopa |
| α-blockers | Prazosin and doxazosin |
| ꞵ-blockers | Acebutolol, alprenolol, atenolol, betaxolol, bisoprolol, bupranolol, carvedilol, esmolol, metipranolol metoprolol, labetalol, levobunolol, nadolol, oxprenolol, pindolol, propranolol, sotalol and timolol |
| Calcium channel blockers |  |
| Dihydropyridines | Amlodipine, barnidipine, benidipine, felodipine, isradipine, lacidipine, lercanidipine, nicardipine, nifedipine, nimodipine, nisoldipine and nitrendipine |
| Non-dihydropyridines | Diltiazem and verapamil |
| Sacubitril | Sacubitril |
| Ivabradine | Ivabradine |
| Diuretics |  |
| Thiazides | Amiloride, bendroflumethiazide, benzylhydrochlorothiazide, clofenamide, clopamide, cyclopenthiazide, hydrochlorothiazide, hydroflumethiazide, indapamide, metolazone, , thiabutazide and trichlormethiazide |
| Loop | Bumetanide, ethacrynic acid and furosemide |
| Potassium-sparing agents | Eplerenone, potassium canrenoate, spironolactone and triamterene |
| Statins | Atorvastatin, fluvastatin, lovastatin, pitavastatin, pravastatin, rosuvastatin and simvastatin |
| Others lipid-lowering agents | Acipimox, alirocumab, bezafibrate, cholestyramine, clofibrate, colestipol, etofibrate, evolocumab, ezetimibe, fenofibrate, gemfibrozil, niacin, niceritrol, nicofuranose, nicomol, probucol and simfibrate |
| Antiplatelets | Abciximab, acetylsalicylic acid, aspirin, cilostazol, clopidogrel, dipyridamole, epoprostenol, eptifibatide, iloprost, prasugrel, selexipag, ticagrelor, ticlopidine, tirofiban and treprostinil |
| Anticoagulants | Heparin, warfarin, urokinase, dalteparin, rivaroxaban, phenindione, streptokinase, nadroparine, enoxaparin, tinzaparin, fondaparinux, dabigatran, apixaban, edoxaban, tenecteplase, protein c and alteplase |
| Nitrates | Nitroglycerin, isosorbide dinitrate, pentaerythritol tetranitrate and isosorbide 5-mononitrate |
| Antiarrhythmics | Adenosine, amiodarone, disopyramide, dronedarone, flecainide, ivabradine, lidocaine, mexiletine, prajmaline, procainamide, propafenone, quinidine, rauwolfia serpentina, rescinnamine, reserpine, sparteine and ubidecarenone |
| Digoxin | Digoxin |
| Pentoxifylline | Pentoxifylline |
| NSAIDs | Aceclofenac, acemetacin, alclofenac, alminoprofen, benzydamine, celecoxib, diclofenac, etodolac, etoricoxib, fenbufen, fenoprofen, flufenamate aluminum, flurbiprofen, glucosamine, ibuprofen, indomethacin, ketoprofen, ketorolac, meclofenamate, mefenamic acid, meloxicam, mepirizole, nabumetone, naproxen, niflumic acid, nimesulide, phenylbutazone, piroxicam, piroxicam, rofecoxib, sulindac, tenoxicam, tiaprofenic acid, tolfenamic acid and tolmetin |
| Steroids | Betamethasone, cortisone, dexamethasone, fludrocortisone, hydrocortisone, methylprednisolone, paramethasone, prednisolone and triamcinolone |
| Pentoxifylline | Pentoxifylline |
| Ketosteril | Calcium 3-methyl-2-oxovaleric acid, calcium-methyl-2-oxovaleric acid, calcium-2-oxo-3-phenylpropionic acid, calcium-3-methyl-2-oxobutyric acid, calcium-DL-2-hydroxy-4-(methylthio)-butyric acid, L-lysine acetate, L-threonine, L-tryptophan, L-histidine, L-tyrosine |
| Proton pump inhibitors | Dexlansoprazole, esomeprazole, lansoprazole, omeprazole, pantoprazole and rabeprazole |
| Anticonvulsants | Brivaracetam, carbamazepine, clonazepam, dipropylacetamide, gabapentin, lacosamide, lamotrigine, levetiracetam, oxcarbazepine, perampanel, phenobarbital, phenytoin, pregabalin, primaclone, rufinamide, tiagabine, topiramate, valproate, vigabatrin and zonisamide |
| Antidepressants | Agomelatine, amitriptyline, bupropion, citalopram, clomipramine, dothiepin, doxepin, duloxetine, escitalopram, fluoxetine, fluvoxamine, imipramine, maprotiline, mianserin, milnacipran, mirtazapine, moclobemide, oxitriptan, paroxetine, sertraline, trazodone, venlafaxine, viloxazine and vortioxetine |
| Antipsychotics | Amisulpride, aripiprazole, brexpiprazole, chlorpromazine, chlorprothixene, clopenthixol, clotiapine, clozapine, droperidol, flupentixol, fluphenazine, haloperidol, lithium, loxapine, lurasidone, methotrimeprazine, moperone, olanzapine, paliperidone, penfluridol, perphenazine, pimozide, pipotiazine, prochlorperazine, quetiapine, risperidone, sulpiride, thioridazine, thiothixene, trifluoperazine, ziprasidone and zotepine |
| Drugs for hyperkalemia and hyperphosphatemia | Polystyrene sulfonate and calcium acetate |

Abbreviation: CKD, Chronic kidney disease; HbA1c, Glycated hemoglobin; GERD, Gastroesophageal reflux disease; AIDS, Acquired immunodeficiency syndrome; NSAIDs, Non-steroidal anti-inflammatory drugs

**eTable 2. Schoenfeld residuals tests for assessing proportional hazard assumptions, by outcomes**

| **Composite renal outcomes** | **Renal replacement** |
| --- | --- |
|   Global test: *p*=0.87 |   Global test: *p*=0.88 |
| **Renal death** | **Hopitalizaion of kidney-related disease** |
|   Global test: *p*=0.96 |   Global test: *p*=0.16 |

**eTable 2. Schoenfeld residuals tests for assessing proportional hazard assumptions, by outcomes (cont’d)**

| **3-point MACE** | **Hospitalization of heart failure** |
| --- | --- |
|   Global test: *p*=0.46 |   Global test: *p*=0.42 |
| **All-cause mortality** | **Hypoglycemia** |
|   Global test: *p*=0.55 |   Global test: *p*=0.49 |

**eTable 3. All baseline characteristics of DPP-4 inhibitor users versus meglitinide users before and after IPTW**

|  | **Before IPTW** | | | | | **After IPTW** | | | | |
| --- | --- | --- | --- | --- | --- | --- | --- | --- | --- | --- |
| **Characteristics** | **DPP-4 inhibitor**  **n=5,028** | | **Meglitinide**  **n=2,243** | | **SMD** | **DPP-4 inhibitor**  **n=5,027** | | **Meglitinide**  **n=2,241** | | **SMD** |
| **Demographics, no. (%)** | | | | | | | | | | |
| Age, mean (SD) ^*^ | 68.78 | (12.34) | 68.51 | (12.21) | 0.02 | 68.69 | (12.34) | 68.76 | (12.18) | -0.01 |
| Sex (male) | 2,508 | (49.88) | 1,152 | (51.36) | 0.09 | 2,525 | (50.22) | 1,122 | (50.05) | 0.01 |
| Cohort entry year | | | | | | | | | | |
| 2013 | 295 | (5.87) | 335 | (14.94) | -0.30 | 435 | (8.65) | 194 | (8.65) | 0.00 |
| 2014 | 466 | (9.27) | 398 | (17.74) | -0.25 | 597 | (11.88) | 268 | (11.94) | 0.00 |
| 2015 | 556 | (11.06) | 319 | (14.22) | -0.10 | 607 | (12.07) | 271 | (12.07) | 0.00 |
| 2016 | 774 | (15.39) | 329 | (14.67) | 0.02 | 760 | (15.11) | 336 | (15.00) | 0.00 |
| 2017 | 897 | (17.84) | 330 | (14.71) | 0.08 | 851 | (16.94) | 388 | (17.32) | -0.01 |
| 2018 | 999 | (19.87) | 289 | (12.88) | 0.19 | 889 | (17.69) | 395 | (17.61) | 0.00 |
| 2019 | 1,041 | (20.70) | 243 | (10.83) | 0.27 | 888 | (17.66) | 390 | (17.42) | 0.01 |
| Monthly income-based insurance premium (NTD) | | | | | | | | | | |
| First quintile | 1,344 | (26.73) | 569 | (25.37) | 0.03 | 1,318 | (26.23) | 577 | (26.23) | 0.01 |
| Second quintile | 497 | (9.88) | 338 | (15.07) | -0.16 | 574 | (11.42) | 255 | (11.42) | 0.00 |
| Third quintile | 909 | (18.08) | 422 | (18.81) | -0.02 | 925 | (18.39) | 423 | (18.39) | -0.01 |
| Forth quintile | 1,252 | (24.90) | 487 | (21.71) | 0.08 | 1,202 | (23.90) | 532 | (23.90) | 0.00 |
| Fifth quintile | 1,026 | (20.41) | 427 | (19.04) | 0.03 | 1,008 | (20.05) | 454 | (20.05) | -0.01 |
| **Measures of health care utilization, no (%)** | | | | | | | | | | |
| Hospital level | | | | | | | | | | |
| Academic medical centers | 1,838 | (36.56) | 808 | (36.02) | 0.01 | 1,833 | (36.47) | 814 | (36.31) | 0.00 |
| Metropolitan hospitals | 2,283 | (45.41) | 1,012 | (45.12) | 0.01 | 2,280 | (45.36) | 1,018 | (45.40) | 0.00 |
| Local community hospitals | 833 | (16.57) | 388 | (17.30) | -0.02 | 840 | (16.71) | 379 | (16.91) | -0.01 |
| Physician clinics | 74 | (1.47) | 35 | (1.56) | -0.01 | 73 | (1.46) | 31 | (1.37) | 0.01 |
| Hospitalizations | | | | | | | | | | |
| Kidney-related | | | | | | | | | | |
| 0 | 4,324 | (86.00) | 1,878 | (83.73) | 0.06 | 4,284 | (85.22) | 1,905 | (85.00) | 0.01 |
| 1 | 586 | (11.65) | 285 | (12.71) | -0.03 | 603 | (11.99) | 273 | (12.19) | -0.01 |
| 2+ | 118 | (2.35) | 80 | (3.57) | -0.07 | 140 | (2.79) | 63 | (2.81) | 0.00 |
| DM-related | | | | | | | | | | |
| 0 | 4,522 | (89.94) | 2,037 | (90.82) | -0.03 | 4,532 | (90.16) | 2,018 | (90.02) | 0.00 |
| 1 | 451 | (8.97) | 186 | (8.29) | 0.02 | 442 | (8.79) | 200 | (8.93) | -0.01 |
| 2+ | 55 | (1.09) | 20 | (0.89) | 0.02 | 53 | (1.05) | 23 | (1.04) | 0.00 |
| CV-related | | | | | | | | | | |
| 0 | 3,848 | (76.53) | 1,661 | (74.05) | 0.06 | 3,815 | (75.89) | 1,708 | (76.19) | -0.01 |
| 1 | 829 | (16.49) | 396 | (17.65) | -0.03 | 842 | (16.74) | 366 | (16.33) | 0.01 |
| 2+ | 351 | (6.98) | 186 | (8.29) | -0.05 | 370 | (7.37) | 168 | (7.48) | 0.00 |
| Other | | | | | | | | | | |
| 0 | 2,937 | (58.41) | 1,331 | (59.34) | -0.02 | 2,951 | (58.69) | 1,321 | (58.93) | 0.00 |
| 1 | 1,223 | (24.32) | 529 | (23.58) | 0.02 | 1,213 | (24.13) | 547 | (24.40) | -0.01 |
| 2+ | 868 | (17.26) | 383 | (17.08) | 0.01 | 863 | (17.18) | 374 | (16.67) | 0.01 |
| OPD visits, mean (SD) ^*^ | | | | | | | | | | |
| Endocrinologist^†^ | 1.81 | (2.81) | 1.64 | (2.68) | 0.06 | 1.76 | (2.78) | 1.77 | (2.72) | 0.00 |
| Nephrologist | 5.72 | (4.65) | 5.79 | (4.67) | -0.02 | 5.77 | (4.66) | 5.82 | (4.62) | -0.01 |
| Cardiologist^†^ | 1.99 | (3.07) | 1.95 | (3.02) | 0.01 | 1.97 | (3.06) | 1.96 | (3.04) | 0.00 |
| Other | 20.82 | (15.84) | 20.85 | (15.87) | 0.00 | 20.83 | (15.84) | 20.95 | (15.83) | -0.01 |
| Emergency visits, mean (SD) ^†^ | 1.56 | (2.16) | 1.53 | (2.05) | 0.01 | 1.55 | (2.16) | 1.55 | (2.06) | 0.00 |
| Number of tests ordered, mean (SD) ^*^ | | | | | | | | | | |
| Serum creatinine | 8.73 | (4.73) | 8.50 | (4.62) | 0.05 | 8.67 | (4.68) | 8.68 | (4.73) | 0.00 |
| Microalbuminuria^†^ | 0.82 | (1.22) | 0.65 | (1.08) | 0.15 | 0.77 | (1.18) | 0.77 | (1.18) | 0.00 |
| HbA1c | 4.02 | (2.11) | 3.78 | (2.11) | 0.11 | 3.95 | (2.09) | 3.96 | (2.18) | -0.01 |
| Lipid | 3.34 | (2.15) | 3.19 | (2.13) | 0.07 | 3.30 | (2.15) | 3.31 | (2.15) | 0.00 |
| Pre-ESRD program | 2,319 | (46.12) | 990 | (44.14) | 0.04 | 2,289 | (45.54) | 1,022 | (45.61) | 0.00 |
| **Proxy indicators of DM severity, no (%)** | | | | | | | | | | |
| Exposure status | | | | | | | | | | |
| New users | 3,697 | (73.53) | 1,674 | (74.63) | -0.03 | 3,713 | (73.86) | 1,646 | (73.45) | 0.01 |
| Persistent combination therapy users | 1,331 | (26.47) | 569 | (25.37) | 0.03 | 1,314 | (26.14) | 595 | (26.55) | -0.01 |
| Duration of previous therapy, mean days (SD) ^*^ | 90.20 | (258.82) | 87.23 | (248.44) | 0.01 | 89.16 | (257.64) | 89.97 | (248.77) | 0.00 |
| **DM medication at cohort entry date** | | | | | | | | | | |
| Level of antidiabetic treatment | | | | | | | | | | |
| One or more insulin | 2,145 | (42.66) | 925 | (41.24) | 0.03 | 2,131 | (42.40) | 954 | (42.57) | 0.00 |
| Two or more classes of non-insulin DM drugs | 257 | (5.11) | 89 | (3.97) | 0.06 | 242 | (4.80) | 111 | (4.96) | -0.01 |
| Other (only study drug) | 2,626 | (52.23) | 1,229 | (54.79) | -0.05 | 2,654 | (52.80) | 1,176 | (52.47) | 0.01 |
| TZD | 173 | (3.44) | 47 | (2.10) | 0.08 | 152 | (3.02) | 66 | (2.94) | 0.00 |
| GLP-1RA^‡^ | 13 | (0.26) | 15 | (0.67) | -0.06 | 12 | (0.24) | 20 | (0.89) | -0.09 |
| Insulin | 2,145 | (42.66) | 925 | (41.24) | 0.03 | 2,131 | (42.40) | 954 | (42.57) | 0.00 |
| Biguanides | 106 | (2.11) | 25 | (1.11) | 0.08 | 91 | (1.80) | 42 | (1.87) | -0.01 |
| AGI | 211 | (4.20) | 92 | (4.10) | 0.00 | 211 | (4.20) | 95 | (4.25) | 0.00 |
| SGLT2i^‡^ | 5 | (0.10) | 4 | (0.18) | -0.02 | 5 | (0.09) | 9 | (0.40) | -0.06 |
| **DM medication within 180 days prior to entry date** | | | | | | | | | | |
| Level of antidiabetic treatment | | | | | | | | | | |
| One or more insulin | 3,018 | (60.02) | 1,276 | (56.89) | 0.06 | 2,978 | (59.24) | 1,334 | (59.54) | -0.01 |
| Two or more classes of non-insulin DM drugs | 676 | (13.44) | 285 | (12.71) | 0.02 | 658 | (13.10) | 289 | (12.89) | 0.01 |
| Other (only study drug) | 1,334 | (26.53) | 682 | (30.41) | -0.09 | 1,390 | (27.66) | 618 | (27.57) | 0.00 |
| TZD | 410 | (8.15) | 129 | (5.75) | 0.09 | 372 | (7.39) | 164 | (7.30) | 0.00 |
| GLP-1RA | 62 | (1.23) | 25 | (1.11) | 0.01 | 61 | (1.21) | 29 | (1.29) | -0.01 |
| Insulin | 3,018 | (60.02) | 1,276 | (56.89) | 0.06 | 2,978 | (59.24) | 1,334 | (59.54) | -0.01 |
| Biguanides | 574 | (11.42) | 231 | (10.30) | 0.04 | 554 | (11.03) | 251 | (11.21) | -0.01 |
| AGI | 630 | (12.53) | 279 | (12.44) | 0.00 | 628 | (12.49) | 284 | (12.69) | -0.01 |
| SGLT2i | 38 | (0.76) | 8 | (0.36) | 0.05 | 32 | (0.64) | 18 | (0.82) | -0.02 |
| aDCSI, mean(SD) ^*^ | 4.41 | (2.34) | 4.24 | (2.38) | 0.07 | 4.35 | (2.37) | 4.34 | (2.32) | 0.01 |
| Hypoglycemia | 725 | (14.42) | 291 | (12.97) | 0.04 | 703 | (13.98) | 311 | (13.89) | 0.00 |
| **Proxy indicators of renal severity, no (%)** | | | | | | | | | | |
| Periods from EPO initiation to cohort entry, mean days (SD) ^*^ | 130.10 | (218.55) | 112.21 | (185.73) | 0.09 | 124.72 | (210.66) | 123.12 | (201.75) | 0.01 |
| Acute kidney disease | 1,083 | (21.54) | 468 | (20.86) | 0.02 | 1,075 | (21.38) | 476 | (21.23) | 0.00 |
| Proteinuria | 171 | (3.40) | 91 | (4.06) | -0.03 | 181 | (3.61) | 87 | (3.88) | -0.01 |
| Disorders of electrolyte | 957 | (19.03) | 468 | (20.86) | -0.05 | 993 | (19.76) | 460 | (20.52) | -0.02 |
| Disorders of fluid balance | 148 | (2.94) | 84 | (3.74) | -0.04 | 161 | (3.20) | 71 | (3.17) | 0.00 |
| Edema | 501 | (9.96) | 330 | (14.71) | -0.14 | 570 | (11.34) | 252 | (11.24) | 0.00 |
| Kidney and urinary stone | 191 | (3.80) | 75 | (3.34) | 0.02 | 187 | (3.72) | 89 | (3.96) | -0.01 |
| **Comorbidities** | | | | | | | | | | |
| **Cardiovascular disease** | | | | | | | | | | |
| Myocardial infarction | 296 | (5.89) | 127 | (5.66) | 0.01 | 290 | (5.76) | 129 | (5.74) | 0.00 |
| Ischemic stroke | 553 | (11.00) | 261 | (11.64) | -0.02 | 561 | (11.15) | 249 | (11.13) | 0.00 |
| Hemorrhage stroke | 77 | (1.53) | 28 | (1.25) | 0.02 | 71 | (1.40) | 27 | (1.21) | 0.02 |
| Other stroke | 677 | (13.46) | 302 | (13.46) | 0.00 | 677 | (13.47) | 303 | (13.54) | 0.00 |
| Heart failure | 1,427 | (28.38) | 624 | (27.82) | 0.01 | 1,410 | (28.04) | 613 | (27.33) | 0.02 |
| Arrhythmia | 402 | (8.00) | 192 | (8.56) | -0.02 | 411 | (8.17) | 182 | (8.10) | 0.01 |
| Peripheral arterial disease | 189 | (3.76) | 97 | (4.32) | -0.03 | 199 | (3.96) | 91 | (4.07) | -0.01 |
| Venous thromboembolism | 56 | (1.11) | 32 | (1.43) | -0.03 | 63 | (1.25) | 27 | (1.20) | 0.00 |
| Coronary revascularization | 193 | (3.84) | 82 | (3.66) | 0.01 | 190 | (3.77) | 85 | (3.77) | 0.00 |
| Hypertension | 4,452 | (88.54) | 2,012 | (89.70) | -0.04 | 4,469 | (88.91) | 1,998 | (89.12) | -0.01 |
| Dyslipidemia | 2,344 | (46.62) | 1,025 | (45.70) | 0.02 | 2,330 | (46.35) | 1,048 | (46.78) | -0.01 |
| **Pulmonary diseases** | | | | | | | | | | |
| Asthma | 280 | (5.57) | 139 | (6.20) | -0.03 | 293 | (5.83) | 131 | (5.84) | 0.00 |
| COPD | 481 | (9.57) | 241 | (10.74) | -0.04 | 501 | (9.96) | 219 | (9.78) | 0.01 |
| Pneumonia | 932 | (18.54) | 422 | (18.81) | -0.01 | 936 | (18.61) | 415 | (18.52) | 0.00 |
| **Psychiatric disorder** | | | | | | | | | | |
| Depression | 234 | (4.65) | 98 | (4.37) | 0.01 | 231 | (4.60) | 109 | (4.84) | -0.01 |
| Anxiety | 390 | (7.76) | 170 | (7.58) | 0.01 | 389 | (7.73) | 179 | (7.97) | -0.01 |
| Schizophrenia^‡^ | 24 | (0.48) | 7 | (0.31) | 0.03 | 23 | (0.46) | 7 | (0.32) | 0.02 |
| **Neurologic disorders** | | | | | | | | | | |
| Dementia | 341 | (6.78) | 140 | (6.24) | 0.02 | 333 | (6.63) | 157 | (7.01) | -0.02 |
| Epilepsy | 72 | (1.43) | 40 | (1.78) | -0.03 | 76 | (1.51) | 33 | (1.47) | 0.00 |
| **Bone and joint disorders** | | | | | | | | | | |
| Fracture | 451 | (8.97) | 202 | (9.01) | 0.00 | 451 | (8.98) | 197 | (8.80) | 0.01 |
| Osteoporosis | 149 | (2.96) | 71 | (3.17) | -0.01 | 154 | (3.05) | 68 | (3.02) | 0.00 |
| Osteoarthritis | 819 | (16.29) | 352 | (15.69) | 0.02 | 814 | (16.18) | 369 | (16.45) | -0.01 |
| **Other diseases** | | | | | | | | | | |
| Anemia | 1,756 | (34.92) | 857 | (38.21) | -0.07 | 1,804 | (35.90) | 809 | (36.12) | 0.00 |
| Thyroid disease | 194 | (3.86) | 69 | (3.08) | 0.04 | 184 | (3.66) | 84 | (3.76) | -0.01 |
| Liver disease | 472 | (9.39) | 189 | (8.43) | 0.03 | 460 | (9.14) | 213 | (9.49) | -0.01 |
| GERD | 599 | (11.91) | 243 | (10.83) | 0.03 | 581 | (11.55) | 257 | (11.48) | 0.00 |
| Autoimmune diseases | 215 | (4.28) | 95 | (4.24) | 0.00 | 213 | (4.24) | 92 | (4.12) | 0.01 |
| Tobacco | 68 | (1.35) | 35 | (1.56) | -0.02 | 72 | (1.43) | 32 | (1.44) | 0.00 |
| Obesity or weight gain^‡^ | 32 | (0.64) | 8 | (0.36) | 0.04 | 31 | (0.62) | 9 | (0.42) | 0.03 |
| Alcohol-related disorder^‡^ | 15 | (0.30) | 7 | (0.31) | 0.00 | 14 | (0.28) | 10 | (0.45) | -0.03 |
| AIDS^‡^ | 3 | (0.06) | 3 | (0.13) | -0.02 | 3 | (0.06) | 5 | (0.22) | -0.05 |
| **Comedications, no (%)** | | | | | | | | | | |
| **Cardiovascular system drugs** | | | | | | | | | | |
| ACEIs | 585 | (11.63) | 262 | (11.68) | 0.00 | 585 | (11.63) | 262 | (11.71) | 0.00 |
| ARBs | 3,038 | (60.42) | 1,270 | (56.62) | 0.08 | 2,978 | (59.25) | 1,339 | (59.73) | -0.01 |
| α-agonists | 42 | (0.84) | 25 | (1.11) | -0.03 | 47 | (0.94) | 20 | (0.91) | 0.00 |
| α-blockers | 1,314 | (26.13) | 614 | (27.37) | -0.03 | 1,329 | (26.44) | 592 | (26.42) | 0.00 |
| ꞵ-blockers | 3,101 | (61.67) | 1,418 | (63.22) | -0.03 | 3,128 | (62.22) | 1,403 | (62.58) | -0.01 |
| Calcium channel blockers | | | | | | | | | | |
| Dihydropyridines | 4,194 | (83.41) | 1,919 | (85.56) | -0.06 | 4,225 | (84.05) | 1,886 | (84.13) | 0.00 |
| Non-dihydropyridines | 401 | (7.98) | 209 | (9.32) | -0.05 | 419 | (8.34) | 183 | (8.18) | 0.01 |
| Sacubitril^‡^ | 39 | (0.78) | 7 | (0.31) | 0.06 | 33 | (0.66) | 13 | (0.56) | 0.01 |
| Ivabradine^‡^ | 36 | (0.72) | 6 | (0.27) | 0.03 | 32 | (0.64) | 6 | (0.26) | 0.03 |
| Diuretics | | | | | | | | | | |
| Loop | 3,717 | (73.93) | 1,719 | (76.64) | -0.06 | 3,763 | (74.85) | 1,679 | (74.91) | 0.00 |
| Thiazides | 1,535 | (30.53) | 718 | (32.01) | -0.03 | 1,565 | (31.14) | 709 | (31.63) | -0.01 |
| Potassium-sparing agents | 696 | (13.84) | 297 | (13.24) | 0.02 | 687 | (13.67) | 309 | (13.80) | 0.00 |
| Lipid-lowering agents | | | | | | | | | | |
| Statins | 2,763 | (54.95) | 1,168 | (52.07) | 0.06 | 2,717 | (54.05) | 1,212 | (54.06) | 0.00 |
| Non-statin agents | 440 | (8.75) | 171 | (7.62) | 0.04 | 421 | (8.38) | 188 | (8.39) | 0.00 |
| Antiplatelets | 2,635 | (52.41) | 1,169 | (52.12) | 0.01 | 2,630 | (52.32) | 1,179 | (52.62) | -0.01 |
| Anticoagulants | 820 | (16.31) | 348 | (15.51) | 0.02 | 808 | (16.07) | 353 | (15.76) | 0.01 |
| Nitrates | 1,666 | (33.13) | 750 | (33.44) | -0.01 | 1,670 | (33.21) | 742 | (33.08) | 0.00 |
| Antiarrhythmics | 447 | (8.89) | 200 | (8.92) | 0.00 | 445 | (8.84) | 192 | (8.57) | 0.01 |
| Digoxin | 126 | (2.51) | 67 | (2.99) | -0.03 | 135 | (2.68) | 60 | (2.66) | 0.00 |
| Anti-inflammatory agents | | | | | | | | | | |
| NSAIDS | 1,990 | (39.58) | 919 | (40.97) | -0.03 | 2,013 | (40.04) | 903 | (40.30) | -0.01 |
| Steroids | 1,693 | (33.67) | 777 | (34.64) | -0.02 | 1,710 | (34.01) | 760 | (33.91) | 0.00 |
| Other medication | | | | | | | | | | |
| Pentoxifylline | 1,918 | (38.15) | 785 | (35.00) | 0.07 | 1,874 | (37.28) | 837 | (37.35) | 0.00 |
| Ketosteril | 889 | (17.68) | 289 | (12.88) | 0.13 | 814 | (16.20) | 354 | (15.77) | 0.01 |
| Proton pump inhibitors | 1,348 | (26.81) | 550 | (24.52) | 0.05 | 1,309 | (26.04) | 580 | (25.86) | 0.00 |
| Anticonvulsants | 908 | (18.06) | 391 | (17.43) | 0.02 | 900 | (17.91) | 406 | (18.09) | 0.00 |
| Antidepressants | 610 | (12.13) | 232 | (10.34) | 0.06 | 587 | (11.67) | 265 | (11.81) | 0.00 |
| Antipsychotics | 563 | (11.20) | 177 | (7.89) | 0.11 | 511 | (10.17) | 228 | (10.16) | 0.00 |
| Drugs for hyperkalemia and hyperphosphatemia | 2,294 | (45.62) | 1,120 | (49.93) | -0.09 | 2,356 | (46.86) | 1,056 | (47.10) | 0.00 |

Abbreviation: DPP-4 inhibitor, Dipeptidyl peptidase 4 inhibitor; IPTW, inverse probability of treatment weighting; SMD, Standardized mean differences; SD, Standard deviation; DM, Diabetes mellitus; CV, Cardiovascular; OPD, Outpatient department; HbA1c, Glycated hemoglobin; ESRD, End-stage renal disease; TZD, Thiazolidinedione; GLP-1RA, Glucagon-like peptide-1 receptor agonists; AGI, α-glucosidase inhibitor; SGLT2i, Sodium-glucose cotransporter 2 inhibitor; aDCSI, Adapted Diabetes Complication; EPO, erythropoietin-stimulating agent; COPD, Chronic obstructive pulmonary disease; GERD, Gastroesophageal reflux disease; AIDS, Acquired Immunodeficiency Syndrome; ACEIs, Angiotensin-converting enzyme inhibitors; ARBs, Angiotensin II receptor blockers; NSAIDS, Non-steroidal anti-inflammatory drugs.

^*^ Continuous variables were treated as restricted cubic splines with 5 knots in the multiple logistic regression model for estimating propensity scores, except for the time period from EPO initiation to cohort entry, which was treated as restricted cubic splines using 3 knots.

^†^Due to the large number of zero values for this variable, we treated it as a categorical variable with three groups (0, 1, or ≥2) in the propensity score model.

^‡^The variable was excluded from the PS model due to its low representation (less than 0.5%) to avoid bias in the PS prediction.

eTable 4. The mean duration and the reasons for truncation during follow-up for DPP-4 inhibitors and meglitinides, by outcomes

| **Outcome** | **Primary composite renal outcome** | | **Renal replacement** | | **Renal death** | |
| --- | --- | --- | --- | --- | --- | --- |
|  | **DPP-4i** | **Meglitinides** | **DPP-4i** | **Meglitinides** | **DPP-4i** | **Meglitinides** |
| **Follow-up time** |  |  |  |  |  |  |
| Mean (SD), days | 149.2 (246.2) | 89.3 (149.3) | 186.0 (256.9) | 112.1 (155.0) | 381.1 (454.4) | 217.7 (302.2) |
| **Censoring reason, %** |  |  |  |  |  |  |
| Outcome occurrence | 65.4 | 64.3 | 46.1 | 43.5 | 6.5 | 5.7 |
| Treatment discontinuation | 15.9 | 14.4 | 23.0 | 22.0 | 43.0 | 44.1 |
| Treatment switching/add-on | 10.0 | 16.0 | 16.0 | 24.7 | 25.4 | 39.5 |
| Death | 2.2 | 2.0 | 6.6 | 5.8 | 3.7 | 2.8 |
| Disenrollment | 5.3 | 3.3 | 7.0 | 3.8 | 16.6 | 6.9 |
| End of study period | 1.2 | 0.1 | 1.3 | 0.1 | 4.8 | 1.0 |

Abbreviations: DPP-4i, dipeptidyl peptidase-4 inhibitor; SD, standard deviation

**eTable 4. The mean duration and the reasons for truncation during follow up for DPP-4 inhibitors and meglitinides, by outcomes (continued)**

| **Outcome** | **Hospitalizations of kidney-related events** | | **3P-MACE** | | **Hospitalization of HF** | |
| --- | --- | --- | --- | --- | --- | --- |
|  | **DPP-4i** | **Meglitinides** | **DPP-4i** | **Meglitinides** | **DPP-4i** | **Meglitinides** |
| **Follow-up time** |  |  |  |  |  |  |
| mean (SD), days | 213.4 (333.4) | 131.4 (228.3) | 371.9 (447.7) | 212.3 (293.5) | 368.8 (447.7) | 210.4 (295.2) |
| **Censoring reason, %** |  |  |  |  |  |  |
| Outcome occurrence | 46.2 | 43.9 | 8.2 | 6,5 | 4.0 | 3.8 |
| Treatment discontinuation | 23.0 | 22.9 | 41.8 | 43.0 | 41.3 | 42.4 |
| Treatment switching/add-on | 13.6 | 21.9 | 24.6 | 38.6 | 24.6 | 38.1 |
| Death | 6.4 | 6.1 | 4.9 | 4.3 | 9.6 | 8.1 |
| Disenrollment | 8.5 | 4.7 | 15.8 | 6.7 | 15.9 | 6.5 |
| End of study period | 2.3 | 0.5 | 4.7 | 1.0 | 4.8 | 1.0 |

Abbreviations: 3P-MACE, 3-point major adverse cardiovascular events; HF, heart failure; DPP-4i, dipeptidyl peptidase-4 inhibitor; SD, standard deviation

**eTable 4. The mean duration and the reasons for truncation during follow-up for DPP-4 inhibitors and meglitinides, by outcomes (continued)**

| **Outcome** | **All-cause mortality** | | **Severe hypoglycemia** | |
| --- | --- | --- | --- | --- |
|  | **DPP-4i** | **Meglitinides** | **DPP-4i** | **Meglitinides** |
| **Follow-up time** |  |  |  |  |
| mean (SD), days | 381.1 (454.4) | 217.7 (302.2) | 361.3 (446.5) | 202.2 (290.2) |
| **Censoring reason, %** |  |  |  |  |
| Outcome occurrence | 10.1 | 8.5 | 7.5 | 9.4 |
| Treatment discontinuation | 43.0 | 44.1 | 40.2 | 39.6 |
| Treatment switching/add-on | 25.4 | 39.5 | 23.4 | 35.8 |
| Death | Outcome of interest | Outcome of interest | 9.1 | 7.9 |
| Disenrollment | 16.6 | 6.9 | 15.4 | 6.3 |
| End of study period | 4.8 | 1.0 | 4.4 | 1.0 |

Abbreviations: DPP-4i, dipeptidyl peptidase-4 inhibitor; SD, standard deviation

**eTable 5. The patient-based number needed to treat for comparative results of DPP-4 inhibitor**

**versus meglitinide**

|  | Cumulative incidence* | | Cumulative incidence difference | NNT^†^ |
| --- | --- | --- | --- | --- |
|  | DPP-4 inhibitor (n=5,028) | Meglitinide (n=2,243) |  |  |
| Composite renal outcome | 0.6601 | 0.7505 | 0.0904 | 11 |
| Renal replacement | 0.5979 | 0.6820 | 0.0841 | 12 |
| Hypoglycemia | 0.0764 | 0.1354 | 0.0590 | 17 |

Abbreviation: DPP-4 inhibitor, dipeptidyl peptidase-4 inhibitor; NNT, number needed to treat

* The cumulative incidence (CI) was obtained from Kaplan-Meier curves, and the NNT was calculated by the formula: NNT=(1/CI_meglitinide_-CI_DPP-4i_).

†NNTs were only calculated for the outcomes with significant differences. The number of NNT represented the number of patients who received DPP-4 inhibitor rather than meglitinide for 1 year to prevent one event.

**eTable 6. Sensitivity analyses of the primary outcome associated with use of DPP-4 inhibitors versus meglitinides**

|  | **Sample size** | **Event, No** | **Crude IR**  **(per 100 PY)** | **Weighted IR**  **(per 100 PY)** | **Crude HR (95% CI)** | **Weighted HR (95% CI)** |
| --- | --- | --- | --- | --- | --- | --- |
| **Primary analyses** |  |  |  |  |  |  |
| DPP-4i | 5,028 | 3,288 | 155.2 | 162.0 | 0.78 (0.73-0.83)* | 0.86 (0.81-0.92)^*^ |
| Meglitinides | 2,243 | 1,378 | 276.0 | 251.6 | 1 (reference) | 1 (reference) |
| **Intention-to-treat analysis** |  |  |  |  |  |  |
| DPP-4i | 5,028 | 3,669 | 181.9 | 189.3 | 0.80 (0.75-0.84)^*^ | 0.88 (0.83-0.93)^*^ |
| Meglitinides | 2,243 | 1,721 | 250.4 | 227.3 | 1 (reference) | 1 (reference) |
| **Use 30-day grace period for continuous use** |  |  |  |  |  |  |
| DPP-4i | 5,028 | 3,136 | 160.8 | 168.0 | 0.78 (0.74-0.83)^*^ | 0.87 (0.81-0.93)^*^ |
| Meglitinides | 2,243 | 1,314 | 280.0 | 255.1 | 1 (reference) | 1 (reference) |
| **Use 90-day grace period for continuous use** |  |  |  |  |  |  |
| DPP-4i | 5,028 | 3,451 | 154.4 | 161.0 | 0.78 (0.74-0.83)^*^ | 0.86 (0.81-0.91)^*^ |
| Meglitinides | 2,243 | 1,446 | 265.0 | 243.2 | 1 (reference) | 1 (reference) |
| **Redefine primary composite outcome** |  |  |  |  |  |  |
| DPP-4i | 5,028 | 3,222 | 150.3 | 156.9 | 0.78 (0.74-0.83)^*^ | 0.87 (0.81-0.92)^*^ |
| Meglitinides | 2,243 | 1,329 | 266.8 | 242.6 | 1 (reference) | 1 (reference) |
| **Competing risk model** |  |  |  |  |  |  |
| DPP-4i | 5,028 | 3,288 | 155.2 | 162.0 | 0.79 (0.75-0.84)^*^ | 0.88 (0.83-0.93)^*^ |
| Meglitinides | 2,243 | 1,378 | 276.0 | 251.6 | 1 (reference) | 1 (reference) |
| **Excluded PS nonoverlapping regions** |  |  |  |  |  |  |
| DPP-4i | 5,028 | 3,328 | 155.2 | 162.0 | 0.78 (0.74-0.83)^*^ | 0.86 (0.81-0.92)^*^ |
| Meglitinides | 2,236 | 1,376 | 275.1 | 251.3 | 1 (reference) | 1 (reference) |
| **Adjusted for unbalanced covariates** |  |  |  |  |  |  |
| DPP-4i | 5,028 | 3,288 | 155.2 | N/A | 0.80 (0.75-0.84)^*^ | 0.83 (0.78-0.88)^*†^ |
| Meglitinides | 2,243 | 1,378 | 276.0 | N/A | 1 (reference) | 1 (reference) |
| **Excluded patients with cancer diagnoses** |  |  |  |  |  |  |
| DPP-4i | 3,929 | 2,680 | 161.3 | 167.4 | 0.80 (0.75-0.86)^*^ | 0.88 (0.82-0.95)^*^ |
| Meglitinides | 1,774 | 1,123 | 277.4 | 253.0 | 1 (reference) | 1 (reference) |
| **Restricted only linagliptin users** |  |  |  |  |  |  |
| Linaglitpin | 3,399 | 2,185 | 163.1 | 168.7 | 0.79 (0.74-0.84)^*^ | 0.87 (0.81-0.93)^*^ |
| Meglitinides | 2,243 | 1,378 | 276.0 | 251.6 | 1 (reference) | 1 (reference) |
| **1:1 PS matching new user design** |  |  |  |  |  |  |
| DPP-4i | 1,420 | 963 | 173.5 | N/A | 0.84 (0.77-0.91)^*‡^ | 0.84 (0.77-0.91)^*‡^ |
| Meglitinides | 1,420 | 915 | 299.5 | N/A | 1 (reference) | 1 (reference) |

Abbreviations: IR, incidence rate; PY, person-year; HR, hazard ratio; CI, confidence interval; DPP-4i, Dipeptidyl peptidase-4 inhibitor; PS, propensity score; N/A, not available;

* *P* < .05.

† HR with 95% CI was estimated by multiple Cox regression model, which adjusted for the number of test orders for microalbuminuria and HbA1c, cohort entry year, monthly income-based insurance premium, edema, antipsychotics, and use of ketosteril.

‡ HR with 95% CI was estimated based on a 1:1 PS matching new-user design, which led to all balanced characteristics between the two groups.

eTable 7. Comparison of the primary outcome between DPP-4 inhibitors and meglitinides, stratified by pre-determined baseline characteristics

|  | **Sample size** | **Event, No** | **Crude IR**  **(per 100 PY)** | **Weighted IR**  **(per 100 PY)** | **Crude HR (95% CI)** | **Weighted HR (95% CI)** | ***p* for interaction** |
| --- | --- | --- | --- | --- | --- | --- | --- |
| **Primary analyses** |  |  |  |  |  |  |  |
| DPP-4i | 5,028 | 3,288 | 155.2 | 162.0 | 0.78 (0.73-0.83)^*^ | 0.86 (0.81-0.92)^*^ |  |
| Meglitinides | 2,243 | 1,378 | 276.0 | 251.6 | 1 (reference) | 1 (reference) |  |
| **New user** |  |  |  |  |  |  | *p*=0.122 |
| DPP-4i | 3,697 | 2,491 | 162.3 | 169.6 | 0.78 (0.73-0.83)^*^ | 0.86 (0.80-0.92)^*^ |  |
| Meglitinides | 1,674 | 1,069 | 318.3 | 282.7 | 1 (reference) | 1 (reference) |  |
| **Persistent combination therapy user** |  |  |  |  |  |  |  |
| DPP-4i | 1,331 | 828 | 127.7 | 132.1 | 0.82 (0.72-0.93)^*^ | 0.87 (0.75-1.00) |  |
| Meglitinides | 569 | 308 | 169.1 | 163.3 | 1 (reference) | 1 (reference) |  |
| **With pre-ESRD program** |  |  |  |  |  |  | *p*=0.209 |
| DPP-4i | 2319 | 1,574 | 150.4 | 154.5 | 0.81 (0.74-0.89)^*^ | 0.91 (0.82-1.00) |  |
| Meglitinides | 990 | 593 | 250.5 | 222.6 | 1 (reference) | 1 (reference) |  |
| **Without pre-ESRD program** |  |  |  |  |  |  |  |
| DPP-4i | 2,709 | 1,754 | 159.5 | 167.4 | 0.75 (0.69-0.81)^*^ | 0.83 (0.76-0.91)^*^ |  |
| Meglitinides | 1,253 | 770 | 298.2 | 274.6 | 1 (reference) | 1 (reference) |  |
| **Use of insulin** |  |  |  |  |  |  | *p*=0.250 |
| DPP-4i | 3,018 | 2,011 | 180.0 | 185.2 | 0.77 (0.71-0.83)^*^ | 0.82 (0.76-0.89)^*^ |  |
| Meglitinides | 1,276 | 824 | 321.1 | 303.8 | 1 (reference) | 1 (reference) |  |
| **Non-use of insulin** |  |  |  |  |  |  |  |
| DPP-4i | 2,010 | 1,328 | 127.7 | 138.9 | 0.78 (0.71-0.85)^*^ | 0.93 (0.84-1.03) |  |
| Meglitinides | 967 | 560 | 228.5 | 193.8 | 1 (reference) | 1 (reference) |  |
| **With hospitalization of renal events** |  |  |  |  |  |  | *p*=0.047 |
| DPP-4i | 704 | 521 | 306.2 | 317.0 | 0.90 (0.78-1.04) | 0.94 (0.80-1.11) |  |
| Meglitinides | 365 | 276 | 376.1 | 355.6 | 1 (reference) | 1 (reference) |  |
| **Without** **hospitalization of renal events** |  |  |  |  |  |  |  |
| DPP-4i | 4,324 | 2,798 | 141.8 | 147.6 | 0.77 (0.72-0.82)^*^ | 0.85 (0.79-0.91)^*^ |  |
| Meglitinides | 1,878 | 1,115 | 259.1 | 236.0 | 1 (reference) | 1 (reference) |  |
| **With history of CV diseases** |  |  |  |  |  |  | *p*=0.802 |
| DPP-4i | 2,323 | 1,526 | 176.3 | 182.5 | 0.77 (0.71-0.85)^*^ | 0.85 (0.76-0.94)^*^ |  |
| Meglitinides | 1,021 | 616 | 307.7 | 285.5 | 1 (reference) | 1 (reference) |  |
| **Without history of CV diseases** |  |  |  |  |  |  |  |
| DPP-4i | 2,705 | 1,800 | 140.6 | 147.4 | 0.78 (0.72-0.84)^*^ | 0.88 (0.80-0.95)^*^ |  |
| Meglitinides | 1,222 | 760 | 253.5 | 224.3 | 1 (reference) | 1 (reference) |  |
| **Use of ACEI/ARB** |  |  |  |  |  |  | *p*=0.657 |
| DPP-4i | 3,247 | 2,129 | 150.7 | 155.2 | 0.78 (0.72-0.84)^*^ | 0.86 (0.79-0.93)^*^ |  |
| Meglitinides | 1,377 | 833 | 260.4 | 236.1 | 1 (reference) | 1 (reference) |  |
| **Non-use of ACEI/ARB** |  |  |  |  |  |  |  |
| DPP-4i | 1,781 | 1,198 | 163.4 | 173.0 | 0.78 (0.71-0.86)^*^ | 0.86 (0.77-0.95)^*^ |  |
| Meglitinides | 866 | 548 | 301.5 | 277.5 | 1 (reference) | 1 (reference) |  |
| **aDCSI>4** |  |  |  |  |  |  | *p*=0.821 |
| DPP-4i | 2,336 | 1,569 | 177.7 | 184.0 | 0.78 (0.71-0.85)^*^ | 0.84 (0.76-0.93)^*^ |  |
| Meglitinides | 971 | 604 | 304.9 | 283.5 | 1 (reference) | 1 (reference) |  |
| **aDCSI≤4** |  |  |  |  |  |  |  |
| DPP-4i | 2,692 | 1,765 | 139.1 | 146.0 | 0.77 (0.71-0.84)^*^ | 0.84 (0.78-0.92)^*^ |  |
| Meglitinides | 1,272 | 780 | 256.0 | 239.3 | 1 (reference) | 1 (reference) |  |

Abbreviations: IR, incidence rate; PY, person-year; HR, hazard ratio; CI, confidence interval; DPP-4i, Dipeptidyl peptidase-4 inhibitor; CV, Cardiovascular; ACEI, Angiotensin-converting enzyme inhibitors; ARB, Angiotensin II receptor blockers; aDCSI, Adapted Diabetes Complication

* *P* < .05.

**
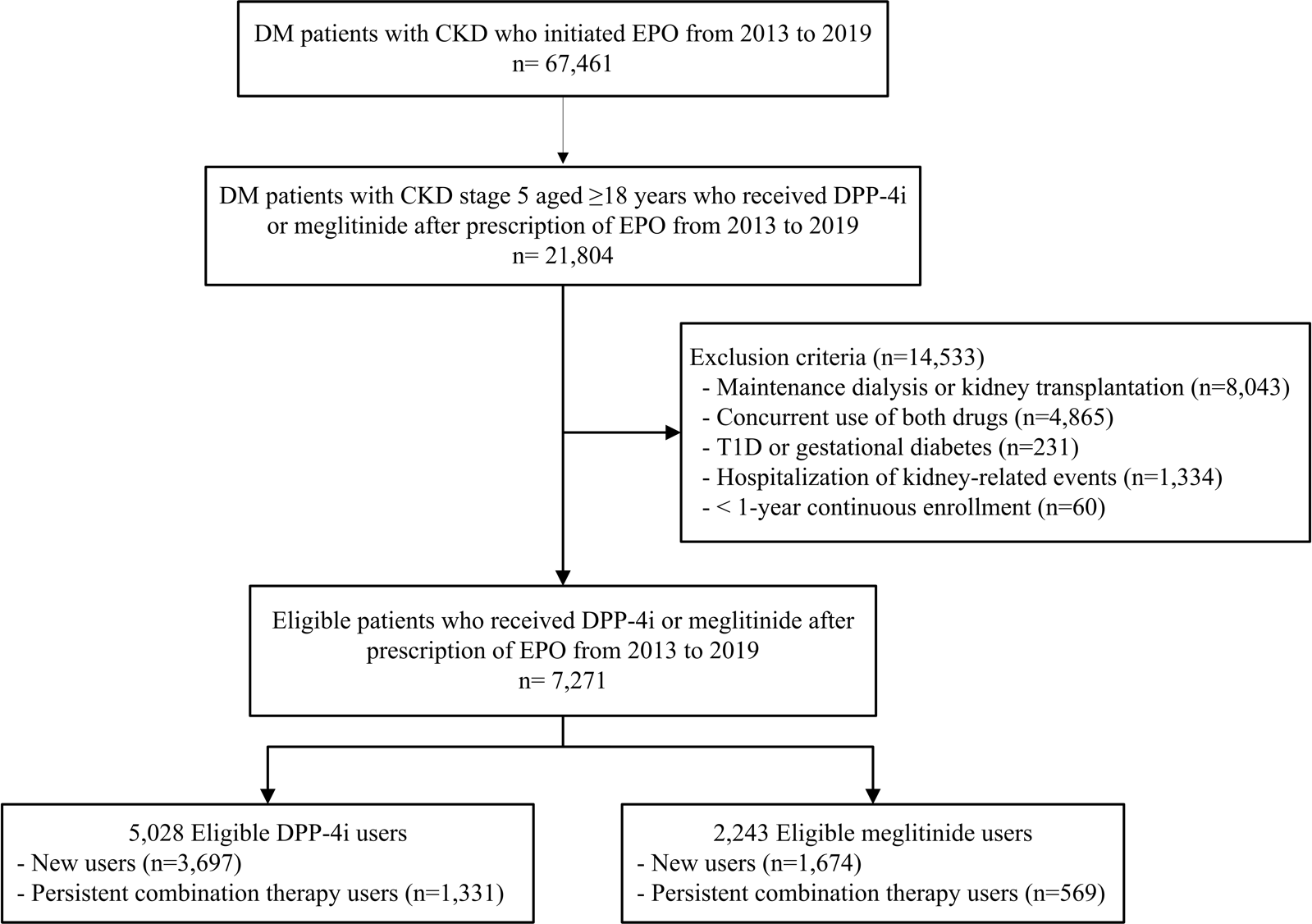
**

**eFigure 1. Flow chart of the eligible study population.**

Abbreviations:DM, Diabetes mellitus; CKD, Chronic kidney disease; EPO, erythropoietin-stimulating agent; DPP-4i, Dipeptidyl peptidase-4 inhibitor

(A)

(B)

(C)

(D)

eFigure 2. Weighted Kaplan Meier survival curves of the primary outcome (A), renal replacement therapy (B), renal death (C) and hospitalization of kidney-related events (D) between users of DPP-4 inhibitors and meglitinide.

Abbreviations: DPP-4i, Dipeptidyl peptidase-4 inhibitor

(A)

(B)

(C)

(D)

eFigure 3. Weighted Kaplan Meier survival curves of 3-point MACE (A), hospitalization of heart failure (B), all-cause mortality (C), hypoglycemia (D) between users of DPP-4 inhibitors and meglitinides.

Abbreviations: DPP-4i, Dipeptidyl peptidase-4 inhibitor, 3P-MACE, 3-point major adverse cardiovascular events

eMethods. Sample size calculation

For the primary analysis, this study was designed with 80% statistical power to detect a 20% difference in the risk of a composite of renal replacement, renal death, and hospitalization for renal events between the two comparison groups. Due to the **lack of data on the incidence rate of renal events among meglitinide users** with stage 5 CKD, we used patients receiving DPP-4 inhibitors as the comparator group to calculate the sample size required for assessing the differences in the renal events in diabetic patients with stage 5 CKD.

We used OpenEpi version 3.01 for sample size calculations based on several key parameters.

First, the type I error was set at 0.05.

Second, using DPP-4 inhibitors as the comparator, we referenced two relevant studies on the incidence rate of renal events among DPP-4 inhibitor users. Among patients with T2DM and primarily early-to-moderate CKD, Pasternak *et al.* compared SGLT2 inhibitors with DPP-4 inhibitors to investigate severe renal composite outcomes, including renal replacement, renal death, and hospitalization for renal events^1^. They documented an incidence rate of 0.62 per 100 person-years for the composite outcome in DPP-4 inhibitor users, with a separate incidence rate of 0.25 per 100 person-years for renal replacement.^1^ On the other hand, Lin *et al.* focused on patients with type 2 diabetes and eGFR <30 mL/min/1.73 m² to examine the risk of renal replacement, finding an incidence rate of 15.9 per 100 person-years in the DPP-4 inhibitor group^2^, which is 63.6 times higher than the rate reported by Pasternak *et al.* for renal replacement.

Given the similarity in patient characteristics between Lin *et al.*’s study^2^ and our study population, we expected the incidence rate of renal replacement in the current study to be comparable to that reported in Lin *et al.*’s study. We also used the incidence rate ratio of renal replacement therapy from Lin *et al.* relative to that in Pasternak *et al*. to estimate the incidence rate of the renal composite outcome in patients with severe CKD based on the incidence rate reported for patients with early-to-moderate CKD. Therefore, an incidence rate of 39.4 per 100 person-years was estimated for renal composite outcomes.

Third, we employed a 1:1 ratio of patients between the study drug group and the control group.

Fourth, the correlation coefficient between the exposure group and the control group was set to be 0 and 0.5, respectively.

Fifth, given that the CARMELINA trial^3^ demonstrated that DPP-4 inhibitors reduce the risk of albuminuria by 16%, we assumed that this reduction in albuminuria risk could translate into a similar reduction in severe renal hard endpoints. Accordingly, we set the effect size at a 20% difference in the renal composite outcome.

Lastly, based on the parameters mentioned above, it is estimated that each group requires 1,946 to 3,926 patients to detect a 20% reduction in the risk of renal outcomes in DPP-4 inhibitors versus meglitinides, with 80% statistical power.

**References**

1. Pasternak B, Wintzell V, Melbye M, et al. Use of sodium-glucose co-transporter 2 inhibitors and risk of serious renal events: Scandinavian cohort study. *BMJ*. Apr 29 2020;369:m1186. doi:10.1136/bmj.m1186

2. Lin Y, Wang TH, Tsai ML, et al. The cardiovascular and renal effects of glucagon-like peptide 1 receptor agonists in patients with advanced diabetic kidney disease. *Cardiovasc Diabetol*. Mar 17 2023;22(1):60. doi:10.1186/s12933-023-01793-9

3. Rosenstock J, Perkovic V, Johansen OE, et al. Effect of Linagliptin vs Placebo on Major Cardiovascular Events in Adults With Type 2 Diabetes and High Cardiovascular and Renal Risk: The CARMELINA Randomized Clinical Trial. *JAMA*. Jan 1 2019;321(1):69-79. doi:10.1001/jama.2018.18269
